# Targeted Manganese doped silica nano GSH-cleaner for treatment of Liver Cancer by destroying the intracellular redox homeostasis

**DOI:** 10.7150/thno.46771

**Published:** 2020-08-02

**Authors:** Hongxia Tang, Chaoqun Li, Yue Zhang, Hongyue Zheng, Ying Cheng, Jingjing Zhu, Xiaojie Chen, Zhihong Zhu, Ji-Gang Piao, Fanzhu Li

**Affiliations:** 1 College of Pharmaceutical Sciences, Zhejiang Chinese Medical University, Hangzhou, 311400, China.; 2 Libraries of Zhejiang Chinese Medical University, Zhejiang Chinese Medical University, Hangzhou, 310053, China.

**Keywords:** glutathione, nanocleaner, sorafenib, ferroptosis, apoptosis

## Abstract

**Background:** Glutathione (GSH), the primary antioxidant in cells, could fight against oxidative stress. Tumor cells display a higher GSH level than normal cells for coping with the hyperoxidative state, which meets the requirements of enhanced metabolism and vicious proliferation. Therefore, the consumption of GSH will lead to cell redox imbalance and impede life activities. Herein, targeted sorafenib (SFB) loaded manganese doped silica nanoparticle (FaPEG-MnMSN@SFB) was constructed, which could destroy the intracellular redox homeostasis by consuming GSH.

**Methods:** In this study, MnMSN was prepared by an optimized one-pot Stober's method for loading SFB, and FaPEG chain was modified on the surface of MnMSN to achieve long circulation and targeted delivery. The anticancer efficacy and mechanism of the designed FaPEG-MnMSN@SFB were assessed both *in vitro* and *in vivo.*

**Results:** FaPEG-MnMSN@SFB exhibited efficient antitumor activity by dual depleting intracellular GSH (the degradation of MnMSN would consume intracellular GSH and the SFB would inhibit the effect of X_c_^-^ transport system to inhibit GSH synthesis). Moreover, disruption of redox balance would lead to apoptosis and reactive oxygen species (ROS)-dependent ferroptosis of tumor cells.

**Conclusion:** Such a GSH-starvation therapeutic strategy would cause multi-path programmed cell death and could be a promising strategy for cancer therapy.

## Introduction

Glutathione, a γ-amide bond and mercapto group-containing tripeptide consisting of glycine, cysteine and L-glutamate, is extensively distributed in mammalian cells and mainly presents in oxidized (GSSG) and reduced (GSH) forms 1. GSH is predominately distributed in the cytosol (about 85%) and is present at lower levels in subcellular organelles, such as mitochondria, nucleus, and endoplasmic reticulum 2. The GSH-centered redox system can control cell growth, immunity and oxidative defense by regulating the redox signaling network 3. For instance, GSH accumulates in the nucleus at an early stage of cell growth; when the cells reach confluence, it is evenly redistributed between nucleus and cytosol 4. Moreover, in cells that are ready to divide, higher nuclear GSH levels are present 3, 5. Mitochondria, the main sites for aerobic respiration and reactive oxygen species (ROS) production, need a certain amount of GSH to maintain the redox balance 6, 7. In the cytosol, GSH-activated glutathione peroxidase 4 (GPx4) could reduce toxic lipid peroxide (PL-PUFA-OOH) to non-toxic polyunsaturated fatty acid (PL-PUFA-OH) and protect the cell membrane from breakage due to the accumulation of PL-PUFA-OOH 8. In summary, GSH participates in many cellular metabolic activities, including maintaining redox balance, mediating signal transduction, and regulating DNA synthesis 9, 10.

Generally, a higher ROS level is required in tumor cells than that of normal cells to inhibit the enhanced metabolism and malignant proliferation 11,12. However, the greater ROS levels can also be offset by the enhanced activity of antioxidative defense system. GSH is the most abundant endogenous antioxidant which could protect tumor cells from a high level of ROS 13. Meanwhile, increasing GSH content has been detected in various tumor cells, which exerts a considerable influence on tumor pathology 14. On the contrary, the reduction or absence of GSH affects the functions and life activities of tumor cells, such as cell division, mitochondrial function, and cell membrane structure 15. Nevertheless, few studies have analyzed the role of "GSH starvation" in suppressing tumors from a holistic perspective. This study aims to explore the effect and mechanism of "GSH starvation" for cancer therapy.

Substantial progress in nanoscience has enabled the availability of an increasing number of options for cancer therapy. Nanomaterials have demonstrated enormous potential in drug encapsulation, cell membrane penetration and tumor-specific drug release through the targeted and EPR effect, which could increase the drug accumulation in tumor cells while decreasing that in normal cells 16, 17. More importantly, nanotechnology endows the nanomaterials with specific functions, such as supplying ROS, consuming GSH, and disturbing the redox balance, thereby playing a substantial role in tumor suppression 18-20. Recently, tumor-microenvironment-responsive biodegradable silica nanoparticles have attracted wide attention, as they can reduce the accumulative toxicity *in vivo* while retaining the advantages of silica materials in drug delivery and controlled release 21-23. And among all kinds of degradable silicon nanoparticles, manganese-doped silica nanoparticle (abbreviated as MnMSN) played its unique characteristics. It could be regarded as the probe for detecting GSH for manganese-oxygen bond (-Mn-O-) in MnMSN was easily reduced by a low GSH level. Decomposition of one molecule of -Mn-O- consumes two molecules of GSH. So, the efficiency of GSH depletion resulting from the degradation of MnMSN would be sufficiently high enough to exhaust the intracellular GSH and break the redox balance of tumor cells. Nevertheless, depleting GSH in cells alone is not sufficient to achieve the "GSH starvation" strategy, as tumor cells can accelerate GSH synthesis through metabolic replenishment.

**Figure 1:**
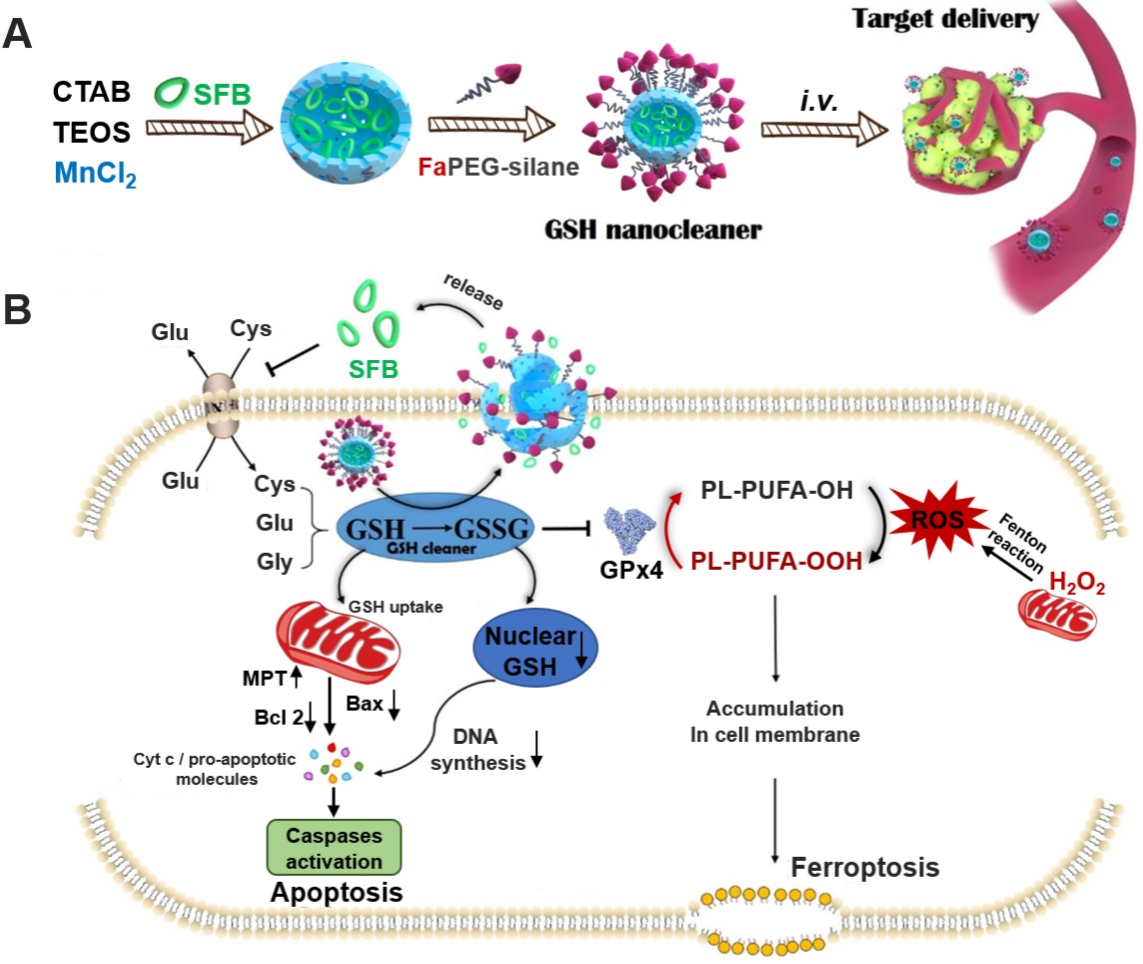
The synthetic route of designed "GSH nanocleaner" (**A**). The mechanisms and pathways of "GSH nanocleaner" induced cell apoptosis and ferroptosis (**B**).

Biosynthesis of GSH is performed by catalysis of glycine, cysteine, and L-glutamate through successive two-step enzyme-catalyzed reactions which are dependent on ATP 24. Among the synthetic materials, most scarce cysteine is acquired by reducing cystine absorbed through the cysteine/glutamate exchange system (X_c_^-^ transport system) to transport cysteine into cells 25. Sorafenib (SFB), a clinically-approved drug, can block X_c_^-^ transport system to inhibit the biosynthesis of GSH (Figure 1B) 26, 27. Thus, the SFB loaded MnMSN (MnMSN@SFB) could exert dual GSH cleaning effects through inhibiting the synthesis of intracellular GSH and the consumption of GSH. Since GSH depletion would lead to more H_2_O_2_ remained in cancer cells 28, 29, •OH produced by Fenton-like reaction of H_2_O_2_ at the presence of Mn^2+^ was also increased, which could improve the chemodynamic therapy catalyzed by Mn^2+^. To further prolong the blood circulation time and improve the tumor-targeting efficiency of nanoparticles, folate grafted PEG (FaPEG) was modified on the outer surface of MnMSN@SFB by silicon hydrolysis reaction. In summary, we constructed a kind of targeted SFB loaded MnMSN (FaPEG-MnMSN@SFB, described as nanocleaner, Figure 1A) as a responsive chemodynamic agent for dual GSH exhaustion (Figure 1B). The anticancer efficacy and mechanism (through different forms of programmed cell death) of the "GSH starvation" strategy were studied both *in vitro* and *in vivo.*

## Methods

### Materials

Cetyltrimethylammonium bromide (CTAB), fluorescein isothiocyanate (FITC), tetraethyl orthosilicate (TEOS), Triton-X-100 lysis buffer and 4′,6-Diamidino-2-phenylindole (DAPI) were purchased from Sigma-Aldrich (USA). Hydrogen peroxide (H_2_O_2_, 30%) and N, N-Dimethylformamide (DMF) were supplied by Sinopharm (Shanghai, China). (3-aminopropyl) triethoxysilane (APTES) and sorafenib tosylate (SFB) were provided by Aladdin (Shanghai, China). mPEG-silane and FaPEG-silane were supplied by Tansh-Tech (China). Manganese chloride tetrahydrate (MnCl_2_·4H_2_O), manganese ion standard solution, silicon ion standard solution and L-Glutathione (GSH) were obtained from Macklin (Shanghai, China). Iron ion standard solution was provided by National Standard Substances Center. Dihydroethidium (DHE) was provided by Abcam. Hydroxyphenyl fluorescein (HPF) was supplied by Shanghai Mao Kang biotechnology Co., Ltd. 3,3',5,5'-Tetramethylbenzidine (TMB) and reduced glutathione (GSH) colorimetric assay kit were purchased from Solarbio Science & Technology Co., Ltd (Beijing, China). Glutathione peroxidase assay kit and Lyso-Tracker Red were supplied by Beyotime (Jiangsu, China). Fer-1 was provided by MedChemExpress (Princeton, USA). Streptomycin sulfate, fetal bovine serum (FBS), penicillin G sodium, RPMI 1640 medium and DMEM medium were purchased from Gibco BRL (USA). 2′,7′-dichlorofluorescin diacetate (DCFH-DA) was obtained from Yuanye Biological Technology (Shanghai, China). C11-BODIPY^581/591^, 5,5′-Dithio bis-(2-nitrobenzoic acid) (DTNB) and 3,3-Dipentyloxacarbocyanine iodide (DIO) were provided by Thermo Fisher (USA). Cy5.5 NHS ester was obtained from Ruixi Biological Technology Co., Ltd (China). Dialysis tube (MW: 3500 D) was supplied by Spectrum Laboratories (USA). All purchased reagents were used without further purification, ultrapure water was produced by the water purification system (PALL Cascade III).

### Cells culture

Normal human liver cells (L02), human umbilical vein endothelial cells (HUVEC), human hepatic carcinoma cells (HepG2), human non-small lung cancer cells (A549) and mouse breast cancer cells (4T1) were supplied by Zhejiang Chinese Medical University (China). L02 and HUVEC cells were cultured in RPMI 1640 medium, while HepG2, A549 and 4T1 cells were cultured in DMEM medium. Both cell culture media were supplemented with 100 units/mL penicillin G sodium, 100 µg/mL streptomycin sulfate and 10% (v/v) FBS at 37 °C with 5% CO_2_ in humidified incubators.

### Animal model

Male SD rats (weighting 200 ± 15 g), male nude mice (3-4 weeks old, approximately 18 g) and New Zealand white rabbits were purchased from the Zhejiang Chinese Medical University Laboratory Animal Research Center. All animal experiments were performed under the protocols approved by the Zhejiang Chinese Medical University Animal Care and Use Committee. Mice were fed in the SPF environment with the temperature of 20 ± 1 °C. The HepG2 tumor-bearing nude mice were acquired by inoculating into their groin with a density of 5×10^6^ cells of the corresponding cells suspension in pH 7.4 PBS.

### Preparation and characterization of MnMSN and FaPEG-MnMSN

Manganese doped silica nanoparticle (MnMSN) was prepared using our original method as previously reported 30. Briefly, 225 mg CTAB was added in 173 mL ethanol/water solution (13%, v/v). Then, 25% ammonium water was added until the pH was 11.5, and the mixture was heated to 75 °C. Subsequently, 1.25 mL TEOS and 5 mL MnCl_2_ solution (containing 110.8 mg MnCl_2_·4H_2_O) were added dropwise and the solution was stirred vigorously for 2 h under 75 °C. After incubation at room temperature for 24 h, the obtained precipitant was collected by ultracentrifugation (12000 rpm, 15 min) and washed for several times with ethanol and water. Finally, the vacuum dried samples were calcined for 6 h at 550 °C to remove the surfactant and obtain MnMSN.

To prepare folate modified long-circulating MnMSN (FaPEG-MnMSN), 40 mg MnMSN was dispersed in 60 mL ethanol and 0.125 mL ammonium water. Subsequently, 10 mL mPEG-silane ethanol solution (containing 40 mg mPEG-silane) and 2 mL FaPEG-silane DMSO solution (containing 2 mg FaPEG-silane) were added dropwise, and the above suspension was stirred for 24 h at 50 °C. Finally, the obtained FaPEG-MnMSN was eluted by ethanol/water (50%, v/v) for three times and vacuum dried for further use.

There are a lot of approaches to characterize both MnMSN and FaPEG-MnMSN. First of all, molecular weight of mPEG-silane and FaPEG-silane was characterized by MALDI-TOF-MS (GCT-Premier, Waters, USA). Transmission electron microscope (FEI Tecnai G2 F20, FEI, USA) was applied to visualize MnMSN and FaPEG-MnMSN. The distribution of Si, O, C, Mn in nanoparticles was obtained by element mapping (Vario EL cube, elementary, Germany). The percentage contents of Si, Mn and O, C, H, N in nanoparticles were determined by inductively coupled plasma emission spectrum (iCAP, 7400, Thermo Fisher Scientific, USA) and elementar analyzer (Vario EL III, Elementar, Germany), respectively. Size and *zeta* potential of the samples were measured via Zetasizer Nano-ZS90 (Malvern Instruments, Malvern, UK). Pore size distribution and surface area of the nanoparticles were calculated by Brunauer Emmett Teller (BET) and Barrett Joyner Halenda (BJH) methods, separately. Nitrogen adsorption-desorption isotherms were obtained by sorptometer (ASAP 2020, Micromeritics, USA) to calculate the specific surface areas of both nanoparticles. Fourier transform infrared (FT-IR) spectrometer (Nicolet IS50, Thermo Fisher Scientific, USA) was used to record the infrared signature of MnMSN and FaPEG-MnMSN. Stability studies were conducted by dispersing MnMSN and FaPEG-MnMSN in PBS (3 mg/mL), the Tyndall phenomenon was observed and the particle sizes were recorded at several time points within 14 days by Malvern Nano-ZS90. Thermogravimetric analysis (TGA, Pyris Diamond, Perkin-Elmer Corporation, USA) was employed to calculate the graft proportion of PEG-silane. The surface morphology of FaPEG-MnMSN was observed by scanning electron microscope (SEM, FEI Nano Nova 450, US).

### Construction and characterization of FaPEG-MnMSN@SFB

To load the SFB, 40 mg MnMSN was dispersed in 10 mL SFB acetone solution (6 mg/mL) and stirred at 25 °C for 8 h. Then, MnMSN@SFB was collected by ultracentrifugation and vacuum dried for further use. Besides, FaPEG-silane was grafted on the surface of MnMSN@SFB as mentioned above to obtain FaPEG-MnMSN@SFB. The drug loading amount (DL%) of SFB formulations was determined by HPLC (1260, Agilent, USA). HPLC conditions: chromatographic column: Agilent-C18 (4.6 × 250 mm, 5 µm); mobile phase: 0.02 mol/L ammonium acetate: methanol = 20:80; column temperature: 40 °C; flow rate: 1.0 mL/min; detection volume: 20 µL; and detection wavelength: 265 nm. The standard curve equation of SFB was *Y*=105.19*X*-3.4218 (*R*^2^ =1) at the linear concentration range from 1 μg/mL to 64 µg/mL. Moreover, DL% was calculated using TGA through heating from 100 to 650 °C.

To estimate the *in vitro* release of SFB, 5 mg MnMSN@SFB or FaPEG-MnMSN@SFB was added in dialysis tubes and placed into centrifuge tubes containing 45 mL PBS solution (pH 7.4, containing 1% Tween 80), then centrifuge tubes were placed in the shaking table at 37 °C, and 1 mL release solution was taken out at 0.25, 0.5, 1, 2, 4 and 8 h (replaced by the equal volume of release medium). After 8 h, the release medium in each group was replaced by 45 mL PBS solution (pH 5.0, GSH=0 mM, containing 1% Tween 80), 45 mL PBS solution (pH 7.4, GSH=10 mM, containing 1% Tween 80) or 45 mL PBS solution (pH 5.0, GSH=10 mM, containing 1% Tween 80), respectively. After that, the remaining release test was conducted as mentioned above (sampling time: 12, 24, 36, 48 and 72 h), and the released SFB was detected by HPLC.

### *In vitro* valence stability of FaPEG-MnMSN in various physiological environments

To investigate the valence stability of FaPEG-MnMSN, 45 mg FaPEG-MnMSN was dispersed into 15 mL various physiological environments (PBS, DMEM medium or serum), then incubated at 37 °C for 24 h. The valence changes of Mn ions in FaPEG-MnMSN were detected by XPS (Thermo Scientific K-Alpha) at 0 h and 24 h.

### *In vitro* decomposition of FaPEG-MnMSN

Decomposition mediums of different GSH concentrations (0 and 10 mM) and pH values (5.0 and 7.4) were applied to imitate the physiological and pathological conditions, then FaPEG-MnMSN was added in dialysis tubes under 37 °C to investigate *in vitro* degradation ability. Manganese and silicon ions decomposed from nanoparticles at predetermined time points were measured by ICP. Moreover, the decomposition solution was also taken out and observed by TEM at 12, 24, 48 and 72 h.

Intracellular decomposition of FaPEG-MnMSN was observed by bio-TEM (JEM 1200EX, Japan). First of all, HepG2 cells (5×10^4^ cells/well) were seeded on a 24-well plate and cultured overnight at 37 °C. Then the medium was exchanged for FaPEG-MnMSN contained culture medium. Finally, bio-TEM images of cells were obtained after frozen section at 24, 48, and 72 h.

### •OH generation of Mn^2+^-driven Fenton-like reaction

TMB assay was performed to monitor the generation of hydroxyl radicals (•OH) through Fenton-like reaction of H_2_O_2_ at the present of Mn^2+^, because TMB could be oxidized by •OH and cause a rapid color change from colorless to blue-green, which has a maximum absorbance at approximately 650 nm. The PBS solution (pH 5.0 or 7.4) containing TMB were mixed with 10 mM Mn^2+^ and different concentrations (0, 0.5, 1, 5, 10 mM) of H_2_O_2_. The generation of •OH was detected by microplate reader at the wavelength between 400 to 800 nm.

### *In vitro* cellular uptake, lysosomal escape and cytotoxicity studies

For cellular uptake experiment, FITC-labeled nanoparticles were prepared as follows: in brief, 100 mg MnMSN was dispersed in 50 mL ethanol, and the mixture was heated to 45 °C. After that, 250 µL water and 250 µL APTES were added successively and dropwise, following by stirred for 8 h at 45 °C. The synthesized aminated MnMSN (MnMSN-NH_2_) was purified by ultracentrifugation and washing. Subsequently, the mixture of 25 mg MnMSN-NH_2_, 1.0 mg FITC, and 5.0 mL DMF was stirred in the dark at 25 °C for 12 h. The obtained FITC-labeled MnMSN (MnMSN@FITC) was collected by ultracentrifugation and eluted with ethanol until the supernatant was colorless. FITC labeled FaPEG-MnMSN (FaPEG-MnMSN@FITC) was synthesized by repeating the steps of grafting FaPEG-silane. After that, HepG2 cells (2×10^5^ cells/well) were seeded into 6-well plates and cultured overnight. Then, cells were treated with culture medium or FITC-labeled nanoparticles contained culture medium (MnMSN@FITC or FaPEG-MnMSN@FITC) with FITC concentration of 3 μmol/L and cultured for another 1 h or 4 h. Subsequently, cells were washed with PBS thrice and collected in flow tubes. Finally, the fluorescence intensity was detected by flow cytometry (Guava Easycyte, Merck Millipore, Germany).

Intracellular localization and lysosomal escape of MnMSN and FaPEG-MnMSN were performed by confocal laser scanning microscope (CLSM, FV 1200, Olympus, Tokyo, Japan). Briefly, HepG2 cells (1×10^5^ cells/well) were seeded into confocal dishes and cultured overnight. Then the medium was exchanged for FITC-labeled nanoparticles contained culture medium (MnMSN@FITC or FaPEG-MnMSN@FITC) with FITC concentration of 5 μmol/L and cultured for another 0.5, 1 or 4 h. Afterwards, cells were rinsed thrice with PBS, stained with Lyso-Tracker Red for 30 min and stained with DAPI for another 15 min. Eventually, the fluorescence signals in each group were observed by CLSM.

The cytotoxicity of the SFB-loaded nanodrugs on L02, HepG2, HUVEC, 4T1 and A549 cells were conducted by MTT assay. All cells (5×10^3^ cells per well) were seeded in a 96-well plate and cultured at 37 °C overnight. Subsequently, cells were incubated with nanocarrier (MnMSN) or SFB formulations (Free SFB, MnMSN@SFB or FaPEG-MnMSN@SFB) at a series of concentrations for 48 h. Afterwards, cells were treated with culture medium containing 5 mg/mL MTT for further 4 h. After the supernatant was discarded, formazan crystal was dissolved in 150 µL DMSO, and the absorbance of every well was measured at a wavelength of 490 nm by the microplate reader.

To investigate the mechanism of nanodrug-induced cell death, HepG2, 4T1 and A549 cells were cultured with GSH (0~10 mM) or Fer-1 (0~40 nM) for 6 h. Then, Free SFB or SFB formulations (containing 20 µg/mL SFB) was added and cultured for 48 h. Subsequently, cells were treated with culture medium containing 5 mg/mL MTT for another 4 h. Eventually, cell viability of each group was measured by the microplate reader.

### GSH concentration, GSH-depletion, Iron contents and GPx4 inhibition effect

To obtain the specific GSH concentration of cancer cells, HepG2 cells (5×10^6^ cells) were collected into a 1.5 mL EP tube. Then, cells were washed twice with PBS, mixed with protein remover, freeze-thawed for three times, and the supernatant was harvested by centrifugation. Afterwards, GSH standard solutions were prepared. And 100 µL supernatant/standard solution was incubated with 100 µL DTNB solution for 20-30 min. The absorbances of above solutions were measured by microplate reader at 412 nm.

To investigate GSH-depletion efficacy of nanodrugs, HepG2 cells (1×10^5^ cells per well) were seeded into a 6-well plate and cultured for 24 h. Next, HepG2 cells were incubated with Free SFB, MnMSN@SFB or FaPEG-MnMSN@SFB for 4 h. After washed with PBS for three times, cells were lysed and the supernatant was harvested by centrifugation. Afterwards, 100 µL supernatant was co-incubated with 100 µL DTNB solution for 20-30 min. Finally, the intracellular GSH content was determined by microplate reader at 412 nm. Untreated cells were used as control group.

For confirming that SFB-loaded nanodrugs could induce ferroptosis of HepG2 cells, the intracellular iron mass changes in response to these nanomedicines were determined. In brief, HepG2 cells (5×10^5^ cells per well) were seeded into 6-well plates and cultured for 12 h. Then, blank medium, Free SFB, MnMSN@SFB or FaPEG-MnMSN@SFB (equivalent dose of SFB=20 µg/mL) was added and treated for 24 h. Next, cells were washed, digested and counted. Finally, the iron contents in each group were measured by ICP-MS (7500ce, Agilent, USA).

Cellular glutathione peroxidase assay kit was applied to investigate GPx4 inhibition effect of nanodrugs. HepG2 cells (1×10^5^ cells/well) were seeded into a 6-well plate and incubated for 12 h. Subsequently, cells were co-incubated with Free SFB, MnMSN@SFB or FaPEG-MnMSN@SFB for 4 h. Afterwards, cell lysates were harvested by centrifugation, and the absorbance was determined by the microplate reader at 340 nm. Untreated cells were used as control group.

### Cell apoptosis and cycle analysis

HepG2 cells (5×10^5^ cells/well) were seeded into 6-well plates and cultured overnight. Subsequently, cells were treated with Free SFB, MnMSN@SFB or FaPEG-MnMSN@SFB for 24 h (equivalent dose of SFB=20 µg/mL). Cells were then washed, trypsinized, and resuspended in Annexin V binding buffer. Afterwards, cells were stained with Annexin V/FITC for 5 min and detected by flow cytometry.

For cell cycle assay, HepG2 cells (5×10^5^ cells per well) were seeded in 6-well plates and treated with Free SFB, MnMSN@SFB or FaPEG-MnMSN@SFB (equivalent dose of SFB=20 µg/mL) for 24 h. Cells were then digested by trypsin and harvested by centrifugation. Subsequently, cells were rinsed with PBS, fixed with 70% ethanol for 12 h. After that, cells were stained with fluorescent solution (0.2% (v/v) Triton X-100, 0.05% PI, 10% RNase) for 30 min and determined by flow cytometry.

### Ferroptosis and apoptosis related proteins expression studies

HepG2 cells (5×10^5^ cells/well) were seeded in a 6-well plate and cultured overnight. Afterwards, cells were incubated with Free SFB, MnMSN@SFB or FaPEG-MnMSN@SFB (containing 20 µg/mL SFB) and cultured for 6 h. Then, cells were lysed with RIPA buffer, and cell lysates were collected by centrifugation and analyzed by using SDS-PAGE gels.

### Intracellular ROS, •OH, •O_2_^-^ and PL-PUFA-OOH generation assay

DCFH-DA as a probe was employed for assessing the intracellular ROS production via CLSM 31. Briefly, HepG2 cells (5×10^5^ cells per well) were seeded in confocal dishes and incubated for 12 h. Then, Free SFB, MnMSN@SFB or FaPEG-MnMSN@SFB at various concentrations (10, 20, and 40 µg/mL of SFB equivalent) was added and treated for 8 h. Subsequently, cells were stained with DCFH-DA (10 µM) for another 20 min. Eventually, CLSM was applied to observe the fluorescence of cells at 488 nm.

It was worth noting that there were many types of ROS, such as superoxide anion (•O_2_^-^), hydrogen peroxide (H_2_O_2_) and hydroxyl radical (•OH). For evaluating the different types of ROS, DHE and HPF were selected as probes to assess the generation of intracellular •O_2_^-^ and •OH, respectively. Briefly, HepG2 cells (5×10^5^ cells per well) were seeded in a 6-well plate and incubated overnight. After that, Free SFB, MnMSN@SFB or FaPEG-MnMSN@SFB at different concentrations (10, 20, and 40 µg/mL of SFB equivalent) were added and treated for 6 h. Cells were then stained with DHE (20 µM) for another 45 min. Eventually, fluorescence microscope was employed to observe the fluorescence of cells at the wavelength of 535 nm. Moreover, to assess the generation of intracellular •OH, HepG2 cells (1×10^5^ cells per well) were seeded into confocal dishes and incubated for 12 h. Then, Free SFB, MnMSN@SFB or FaPEG-MnMSN@SFB at different concentrations (10, 20, and 40 μg/mL of SFB equivalent) were added, meanwhile HPF (10 µM) was added to capture generative intracellular •OH, following by co-incubation for 6 h. Eventually, CLSM was used to observe the fluorescence of cells at 490 nm.

For intracellular PL-PUFA-OOH generation assay, HepG2 cells (5×10^5^ cells/well) were seeded into 4-chamber dishes and cultured overnight. Free SFB, MnMSN@SFB or FaPEG-MnMSN@SFB with various concentrations (10, 20, and 40 µg/mL of SFB equivalent) was added and incubated for further 6 h. Subsequently, fluorescent probe (C11BODIPY^581/591^, 2 µM) was added and stained for another 30 min. Ultimately, fluorescence signals in each group were observed by CLSM at a wavelength of 488 nm.

### Observation of cell membrane morphology after treated with GSH nanocleaner

Morphological changes of cell membranes resulted from GSH nanocleaner were observed by CLSM. In brief, HepG2 cells (5×10^5^ cells/well) were seeded into the confocal dishes and incubated for 12 h. Cells were then cultured with DIO (1 mM) for 3 min. Afterwards, the supernatant was removed and Free SFB, MnMSN@SFB or FaPEG-MnMSN@SFB with various concentrations (10, 20, and 40 µg/mL of SFB equivalent) was added and incubated for 12 h. The morphological changes of cell membranes were finally observed by CLSM at 488 nm.

### Pharmacokinetic study

Plasma pharmacokinetic study was performed in SD rats. Briefly, SD rats were arbitrarily allocated into three groups (n=6), and SFB formulations (10 mg SFB equivalent/kg) were administered via tail vein injection. Blood samples (0.5 mL) were harvested via the orbital vein and placed in heparinized tubes at the predetermined times. The blood samples were centrifuged to obtain plasma. The SFB concentration in the plasma was measured by HPLC method, and itraconazole was selected as internal standard.

### *In vitro/vivo* tumor homing and T1-weighted MRI imaging studies

*In vivo* fluorescence imaging was carried out to observe the tumor homing effect of nanoparticles* in vivo*. First of all, Cy5.5 labeled nanoparticles were prepared as follows: briefly, the mixture of 2 mg MnMSN-NH_2_, 0.3 mg Cy5.5-NHS ester, and 2 mL DMF was stirred in dark at 25 °C for 6 h. The Cy5.5 labeled MnMSN was collected by ultracentrifugation and purified by ethanol/water (3:1, v/v) for five times, then vacuum dried for further use. Cy5.5 labeled FaPEG-MnMSN was obtained through repeating the steps of grafting FaPEG-silane. Afterwards, Cy5.5 labeled nanoparticle (Cy5.5 labeled MnMSN or Cy5.5 labeled FaPEG-MnMSN) at a dose of 3.5 mg/mL (0.2 mL) was injected into tumor-bearing mice via tail vein injection. *In vivo* biodistribution and tumor homing effect of the nanoparticles were monitored and recorded at 1, 2, 4, 8, 12 and 24 h after injection. After injection for 24 h, the isolated major organs of mice were excised and the *in vitro* tissue fluorescence-distribution images were also recorded.

*In vitro* T1-weighted MRI imaging was conducted to investigate the feasibility of decomposition and MRI imaging of nanoparticles. Various concentrations of FaPEG-MnMSN (equivalent Mn concentration of 0, 0.008, 0.032, 0.064, 0.096, and 0.16 mM) were dispersed in different pH (7.4 or 5.0) with/without GSH (10 or 0 mM) solutions and reacted for 24 h to release Mn^2+^. The MRI images were recorded by using the 1.5-T unit MRI system (GE Signal HDxt; GE Healthcare, Little Chalfont, UK).

*In vivo* MRI imaging was taken to explore the tumor homing and tumor diagnosis effects of nanoparticles* in vivo*. 0.2 mL MnMSN or FaPEG-MnMSN dispersion (equivalent Mn concentration of 1.5 mg/kg) was injected into tumor-bearing mice via tail vein, then the MRI signals were recorded at 1, 4, 8, 12 and 24 h after injection by 1.5-T unit MRI system.

*In vivo* tumor-homing effect was also evaluated by measuring the concentration of Mn element on the tumor site and vital organs via ICP-MS. The HepG2 tumor-bearing mice with tumor volume of approximately 200 mm^3^ were arbitrarily allocated into 2 groups (n=6). After that, MnMSN or FaPEG-MnMSN dispersion (equivalent Mn concentration of 3.5 mg/kg) was injected into tumor-bearing mice by tail vein. 4 h later, the mice were executed, tumors and vital organs were harvested, then lysed by the mixture of nitric acid and H_2_O_2_, assessed by ICP-MS.

### *In vivo* anti-tumor and biocompatibility assay

The anti-tumor efficiency of the SFB-loaded nanoparticles was performed as follows. In brief, the HepG2 tumor-bearing mice with tumor volume of approximately 100 mm^3^ were arbitrarily allocated into 5 groups (n=11). Then, FaPEG-MnMSN@SFB, MnMSN@SFB, Free SFB (10 mg SFB equivalent/kg), FaPEG-MnMSN, or Saline was intravenously injected every 2 days. The tumor-bearing mice were weighed and measured (length and width of tumor) followed by each injection (n=6). On 15^th^ day, two mice of each group were executed and major organs were harvested for histopathology analyses with hematoxylin and eosin (H&E) staining. Moreover, the tumor tissues were collected for H&E, TUNEL, and Ki67 staining to evaluate the tissue necrosis, cell apoptosis and proliferation. Meanwhile, three mice of each group were executed and tumors were harvested to measure GSH and GPx4 levels. Here, reduced glutathione (GSH) colorimetric assay kit and glutathione peroxidase assay kit were employed to determine the GSH content and GPx4 activity of tumor tissues, respectively. The remaining six mice were used to monitor the survival rates. The survival curve was drawn and analyzed by Kaplan-Meier analysis. The tumor volume was calculated as the following equation: V_tumor_ = *a*×*b*^2^/2 (*a* and *b* represents the length and width of the tumor, separately).

### Biosafety of nanocleaner

In brief, the hemocompatibility of designed nanocleaner was investigated by using fresh anticoagulant blood which was isolated from New Zealand rabbits. Subsequently, 2% (v/v) red blood suspension was obtained by washing and diluting fresh anticoagulant blood with saline. Then, various concentrations (0.01 ~ 2 mg/mL) of MnMSN and FaPEG-MnMSN were incubated with red blood suspension at 37 °C for 1 h. Eventually, supernatant of each sample was separated to determine the absorbance by applying the microplate reader at 414 nm.

### Statistical analysis

Data was expressed as mean ± standard deviation (SD). Statistical significance in the mean values was evaluated by one-way ANOVA by SPSS software (version 20.0, USA). In all studies, *P* < 0.05 was considered to be statistically significant, and *P* < 0.01 was regarded as extreme significance.

## Results and Discussion

### Preparation and characterization of FaPEG-MnMSN

**Figure 2:**
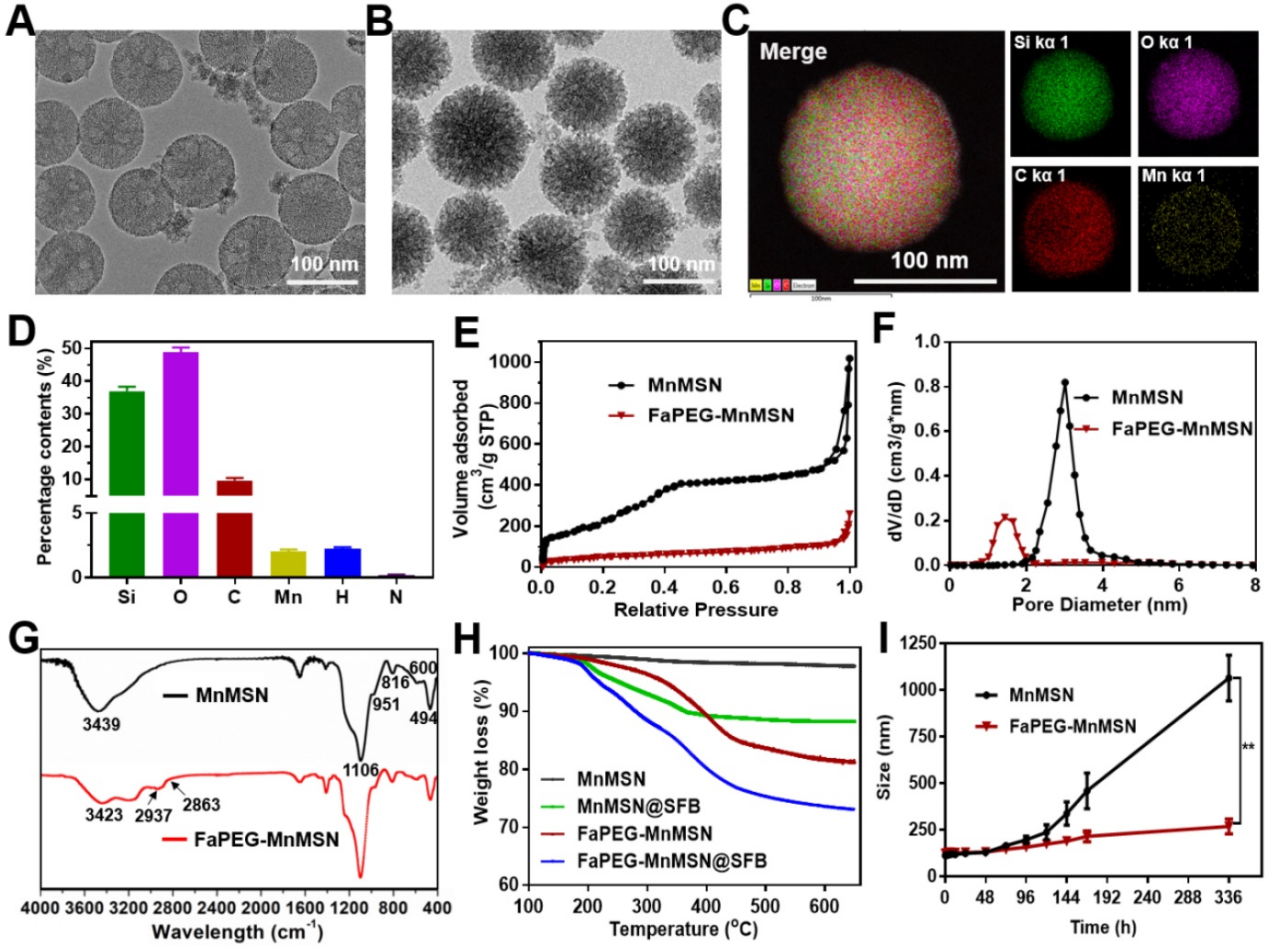
TEM images of MnMSN (**A**) and FaPEG-MnMSN (**B**). Element mappings of FaPEG-MnMSN (**C**). Percentage content of Si, O, C, Mn, H and N in FaPEG-MnMSN (**D**). N_2_ adsorption-desorption isotherms (**E**), pore size distributions (**F**), and FT-IR spectra (**G**) of MnMSN and FaPEG-MnMSN. TGA curves of drug carriers and SFB formulations (**H**). Dynamic light scattering profiles of MnMSN and FaPEG-MnMSN in PBS for 14 days (**I**). **P < 0.01 vs MnMSN group.

MnMSN was prepared by an optimized one-pot Stober's method referred to our previous work 30. In brief, under the alkaline environment (pH=11.5), TEOS and MnCl_2_·4H_2_O were added dropwise and successively to CTAB solution, after which MnMSN was acquired. In the process of reaction, the -Mn-O- bonds formed uniformly in the mesoporous structure during the hydrolysis of TEOS. As presented in Figure 2A and S1, the resultant MnMSN exhibited spherical morphology, uniform particle size and favorable dispersity, with diameter of 101.40 ± 0.36 nm (PDI 0.074 ± 0.014) and *zeta* potential of -23.4 ± 0.2 mV. The elemental analysis results of MnMSN indicated that Mn was uniformly presented in the nanoparticles, and the content of Si, O and Mn was 43.59%, 53.56% and 2.58%, separately (Figure S3).

Considering the fact that MnMSN was unstable and easily aggregated due to high surface energy, as well as to further improve the dispersity, prolong blood circulation time and achieve active targeting function of nanocarriers, folate-modified PEG (FaPEG) was thus linked to the surface of the MnMSN (FaPEG-MnMSN). The successful grafting of folate on the PEG chain was confirmed by MOLDI-TOF-MS (Figure S4). As observed from Figure S1, after grafting with FaPEG, the particle size of FaPEG-MnMSN increased to 122.67 ± 2.98 nm (PDI 0.132 ± 0.003), and the *zeta* potential was changed to -19.3 ± 0.1 mV. By comparison with TEM images (Figure 2A-B), the outer surface of nanoparticles was converted from smooth to rough owing to the modification of FaPEG with a thickness of approximately 20 nm. Meanwhile, the elemental analysis results of FaPEG-MnMSN confirmed that the content of Si, O, C and Mn was changed to 36.96%, 48.76%, 9.63% and 2.05%, respectively (Figure 2C-D).

To verify the end-capped effect of FaPEG chains, the pore diameter distributions and N_2_ adsorption-desorption isotherms of carriers were measured. As shown in Figure 2E-F, MnMSN displayed the type IV isotherm pattern with the pore size of about 3.01 nm, indicating their ordered and regular mesoporous structure. Furthermore, the pore of MnMSN was encapsulated by the FaPEG layer; this was reflected by the marked reduction of specific surface area and cumulative pore volume from 1017.59 m^2^/g and 0.84 cm^3^/g for MnMSN to 259.55 m^2^/g and 0.28 cm^3^/g for FaPEG-MnMSN, separately. Compared with the FT-IR spectrum of MnMSN, the additional peaks appeared at 2937 cm^-1^ and 2863 cm^-1^ caused by the existence of methyl and methylene in FaPEG chains (Figure 2G). Moreover, the broad peak at 3423 cm^-1^ became weaker, which could be attributed to the formation of -Si-O- bonds by Si-OH after FaPEG grafting. Furthermore, the TGA results demonstrated that FaPEG was grafted successfully on the surface of MnMSN, which could be reflected by the additional weight loss of 15.63% during 200-450 °C compared with that of MnMSN (Figure 2H).

### Dispersion stability, GSH-depleting ability and degradability of FaPEG-MnMSN

Mesoporous materials were generally unstable and easily aggregated caused by the high surface energy 32, 33. Thus, appropriate surface modification of MnMSN is necessary for the delivery of anti-tumor drugs. The modification of PEG would enhance the dispersity and compatibility of nanocarriers, resulting from the formation of nonspecific steric hindrance between nanoparticles. Meanwhile, the steric hindrance would prevent the combination of serum proteins with nanoparticles to achieve the effect of prolonged blood circulation time 34-36. The dispersion stability experiments were conducted by observing the Tyndall phenomenon and monitoring the size variation of MnMSN and FaPEG-MnMSN in PBS, respectively. In Figure S5 and 2I, in the MnMSN group, black precipitate appeared at the bottom and its average diameter increased to above 1000 nm being left to stand for 14 days. By contrast, the particle size was relatively stable and uniform in the FaPEG-MnMSN group without a significant change. In general, the graft of FaPEG significantly improved the physical stability of MnMSN.

XPS data were obtained to evaluate the changes of valence states of Mn in nanocarriers after incubation with PBS, DMEM medium or serum for 24 h. Figure S6 showed the Mn 2p spectrum of the FaPEG-MnMSN, and two major peaks at 654 and 642 eV were attributed to Mn 2p_1/2_ and Mn 2p_3/2_, separately 37-39. Then, the spectra of Mn^2+^, Mn^3+^ and Mn^4+^ were acquired through peak fitting to fit Mn 2p_3/2_, and the content ratios of various valence states of Mn were calculated by comparison of peak areas. After multi-peak-resolution process, the valence states of Mn in FaPEG-MnMSN were almost unchanged, indicating that the prepared nanocarriers were stable in various physiological environments.

**Figure 3:**
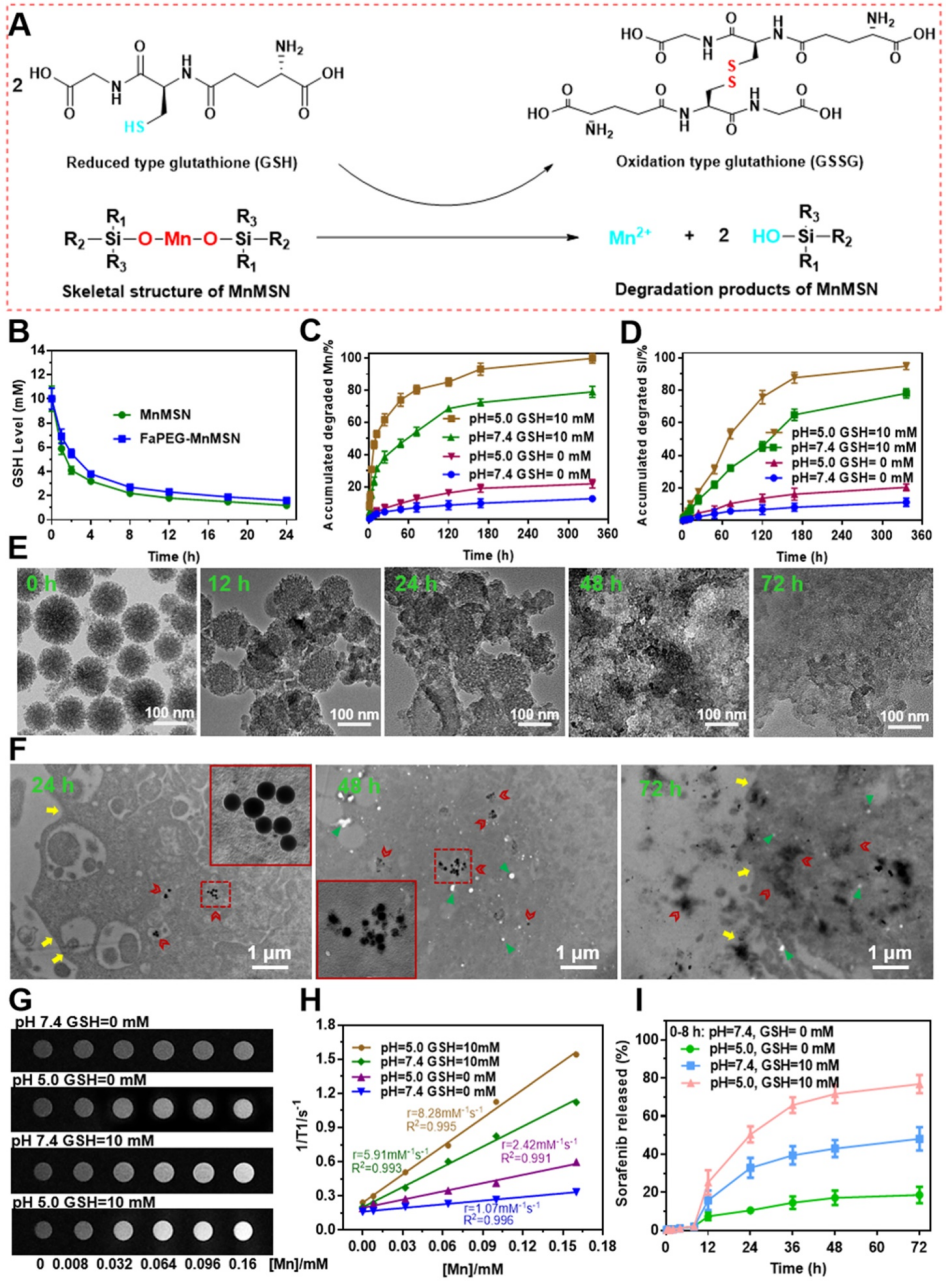
The mechanism of -Mn-O- bonds in MnMSN degraded by GSH (**A**). GSH levels of the solutions after incubated with MnMSN or FaPEG-MnMSN (**B**). Accumulated degraded profiles of Mn (**C**) and Si (**D**) from FaPEG-MnMSN in PBS at various pH with/without GSH. TEM images of FaPEG-MnMSN during degradation for 72 h (**E**). Intracellular biodegradation behaviors of FaPEG-MnMSN after incubated with HepG2 cells for 24, 48, and 72 h (**F**). T1-weighted MRI (**G**) and T1 relaxivity (**H**) of FaPEG-MnMSN in various pH with/without GSH. Cumulative SFB release from FaPEG-MnMSN@SFB in PBS at various pH with/without GSH (**I**).

To investigate the GSH-depleting capacity of MnMSN and FaPEG-MnMSN, the GSH levels after incubation with nanocarriers were monitored in this study. In Figure 3B, the GSH content of MnMSN and FaPEG-MnMSN was reduced by 71.25% and 64.13% at 4 h and by 93.71% and 91.80% after 24 h. The GSH depleting rate of MnMSN was faster than that of FaPEG-MnMSN in the first 4 h for the modification of PEG chains.

Previous researches have evidenced that -Mn-O- bond is sensitive to acidic and reductive environments, which is the representative characteristics of tumor microenvironment 22, 40. As presented in Figure 3A, GSH can reduce the -Mn-O- bonds in MnMSN to Mn^2+^, meanwhile GSH was oxidized to GSSG. Based on the above cognitions, the degradation ability of FaPEG-MnMSN under a variety of GSH and pH conditions was evaluated by using TEM observation and ICP measurement. As presented in Figure 3C-D, the accumulated degraded Mn and Si was 12.77 ± 2.13% and 11.18 ± 3.23% in physiological environment (GSH=0 mM, pH=7.4) after 14 days separately, demonstrating that FaPEG-MnMSN was relatively stable in the normal environment. With the increase of GSH concentration and the decrease of pH, the degradation of FaPEG-MnMSN was enhanced accordingly, reflected by the increase of dissolved Mn and Si. It is worth noting that FaPEG-MnMSN could be degraded either at neutral pH with GSH or at a mildly acidic conditions without GSH, and GSH exhibited a stronger degradation-promoting effect. The accumulated amounts of released Mn and Si reached maximum percentage of 99.86 ± 3.05% and 94.92 ± 3.48% under mildly acidic conditions with GSH (GSH=10 mM, pH=5.0) after 14 days, revealing that the synthetic FaPEG-MnMSN could be degraded rapidly in the tumor microenvironment. In addition, the tendency for accumulated release of Si ions was slower than that of Mn ions in that the -Mn-O- bonds were reduced by GSH directly to obtain Mn^2+^, but the release of Si ions required the further decomposition of -Si-O- bonds. In Figure S7, FaPEG-MnMSN dispersion gradually turned to colorless in PBS with 10 mM GSH during 48 h, while there was no significant change in PBS without GSH. The order of degradation rates of all groups was as follows: A1 (pH=5.0, GSH=10 mM) > A2 (pH=7.4, GSH=10 mM) > A3 (pH=5.0, GSH=0 mM) > A4 (pH=7.4, GSH=0 mM). Furthermore, the degradation process of FaPEG-MnMSN in different conditions was recorded visually by TEM images in Figure 3E and S8. Under acidic conditions with GSH, the border of the FaPEG-MnMSN became hazy at 12 h. After 48 h, the skeleton of nanoparticles collapsed, and only a little fragment remained after incubation for 72 h. However, the structure of FaPEG-MnMSN remained comparatively complete under conditions of pH 7.4 without GSH for 7 days (Figure S8).

To further investigate the intracellular degradation capacity of the designed FaPEG-MnMSN, biological TEM was used to observe the morphological variation of nanoparticles after incubation with HepG2 cells for different times. As displayed in Figure 3F, FaPEG-MnMSN would be phagocytosed by HepG2 cells, followed by accumulation in the cytoplasm at an early stage. On day 2, FaPEG-MnMSN was degraded and many fragments could be found in the cytoplasm. After 72 h, hardly any complete nanoparticles were found and only residue remained. All phenomena confirmed that FaPEG-MnMSN could be degraded in HepG2 cells. Collectively, the intracellular degradation results of FaPEG-MnMSN were in accordance with the *in vitro* degradation results, demonstrating that the slightly acidic and GSH-enriched environment of tumor cells could trigger the breakage of FaPEG-MnMSN.

### MRI imaging feature of FaPEG-MnMSN and Fenton catalytic ability of Mn^2+^

For multi-angle investigating the degradation characteristics of designed FaPEG-MnMSN, a 1.5 T human clinical MRI scanner was used to analyze T1-weighted contrast enhancement, based on the fact that Mn^2+^ released from FaPEG-MnMSN would enhance the r1 relaxivity. In Figure 3H, the r1 relaxivity was 1.07 mM^-1^s^-1^ in neutral environment (pH=7.4, GSH=0 mM), while in mildly acidic environment (pH=5.0, GSH=0 mM) was 2.42 mM^-1^s^-1^, indicating that FaPEG-MnMSN could be degraded under acidic conditions. In the presence of GSH, the r1 relaxivity increased enormously from 1.07 mM^-1^s^-1^ to 5.91 mM^-1^s^-1^ (pH=7.4, GSH=10 mM). Furthermore, the maximum r1 relaxivity was achieved after incubation under acidic conditions with GSH (pH=5.0, GSH=10 mM). Taken together, these results indicated that FaPEG-MnMSN, as a stimuli-responsive T1 MRI contrast agent, played potent roles in tumor imaging.

Interestingly, the dissolved Mn^2+^ could catalyze H_2_O_2_ to generate •OH (a kind of reactive oxygen species) through Fenton reaction (Figure S9-S10). Considering that TMB could be oxidized by •OH to obtain blue-green products, the generation of •OH was measured by TMB assay to assess the catalytic capability of Mn^2+^ in producing •OH. As displayed in Figure S9-S10, neither H_2_O_2_ nor Mn^2+^ alone had obvious color changes or detectable absorbance increases of TMB solutions after incubated for 3 days. In addition, as could be seen in Figure S9, more •OH was produced under acidic conditions (pH 5.0) than under neutral ones (pH 7.4), indicating that the acidic conditions were beneficial for Mn^2+^ to catalyze H_2_O_2_ to generate •OH. Meanwhile, the absorbance of TMB solution was positively related to the concentration of H_2_O_2_ (Figure S10A). The maximum absorbance of mixed solution appeared at approximately 24 h after incubation (Figure S10B). Collectively, all the above results proved that Mn^2+^ exhibited favorable catalytic activity for the generation of •OH from H_2_O_2_ in an acidic environment. The generation of •OH and depletion of GSH will act synergistically to accelerate destruction of the redox balance.

### Drug loading and releasing study

Due to the particular mesoporous structure, large pore volume and intelligent degradation ability, MnMSN@SFB or FaPEG-MnMSN@SFB exhibited high drug loading and stimuli-responsive releasing capacity. The DL% of MnMSN@SFB and FaPEG-MnMSN@SFB was 8.98 ± 0.86% and 8.04 ± 1.03% respectively, which was determined by HPLC method. In order to improve the reliability of the detection results, the DL% of MnMSN@SFB (9.45%) and FaPEG-MnMSN@SFB (8.18%) were also calculated by TGA method, reflected by the weight loss at 200-400 °C (Figure 2H). Drug loading mechanism mainly depended on the coordinate bonds and Van der Waals forces between the SFB and MnMSN/FaPEG-MnMSN.

To investigate the stimuli-responsive release capability of MnMSN@SFB and FaPEG-MnMSN@SFB, *in vitro* release experiment was conducted in various pH and GSH conditions by changing the release medium. As presented in Figure S11 and 3I, few SFB was released from MnMSN@SFB (2.18 ± 0.55%) or FaPEG-MnMSN@SFB (2.07±0.58%) in neutral condition (pH=7.4, GSH=0 mM) during 8 h, demonstrating that nanodrugs were stable under physiological environment. The release rate could be accelerated by increasing GSH concentration or decreasing pH value, resulting from the degradation of nanocarriers. That is, MnMSN@SFB or FaPEG-MnMSN@SFB was sensitive to both high GSH and low pH. The cumulative release rate of MnMSN@SFB under acidic conditions (pH=5.0, GSH=0 mM) and GSH containing conditions (pH=7.4, GSH=10 mM) was 25.66 ± 5.33% and 68.73 ± 8.93% separately during 3 days, indicating that GSH played a more critical role in degrading nanocarriers than H^+^. Under the simulative tumor microenvironment (pH=5.0, GSH=10 mM), the cumulative release of SFB from MnMSN@SFB peaked at 90.42 ± 5.33% at 72 h, whereas in FaPEG-MnMSN@SFB group was 76.75 ± 8.36% (Figure 3I), which confirmed that modification of FaPEG further achieved sustained release effect. Potentially, the unique properties of FaPEG-MnMSN@SFB to maintain stability in the physiological environment while stimuli-responsive releasing in the tumor microenvironment made it a superior carrier for the delivery of anti-tumor drugs, which could reduce side effects of anticancer drugs on normal tissues but ensure accumulation of the drugs at tumor site.

### Cell uptake and lysosomal escape studies

**Figure 4:**
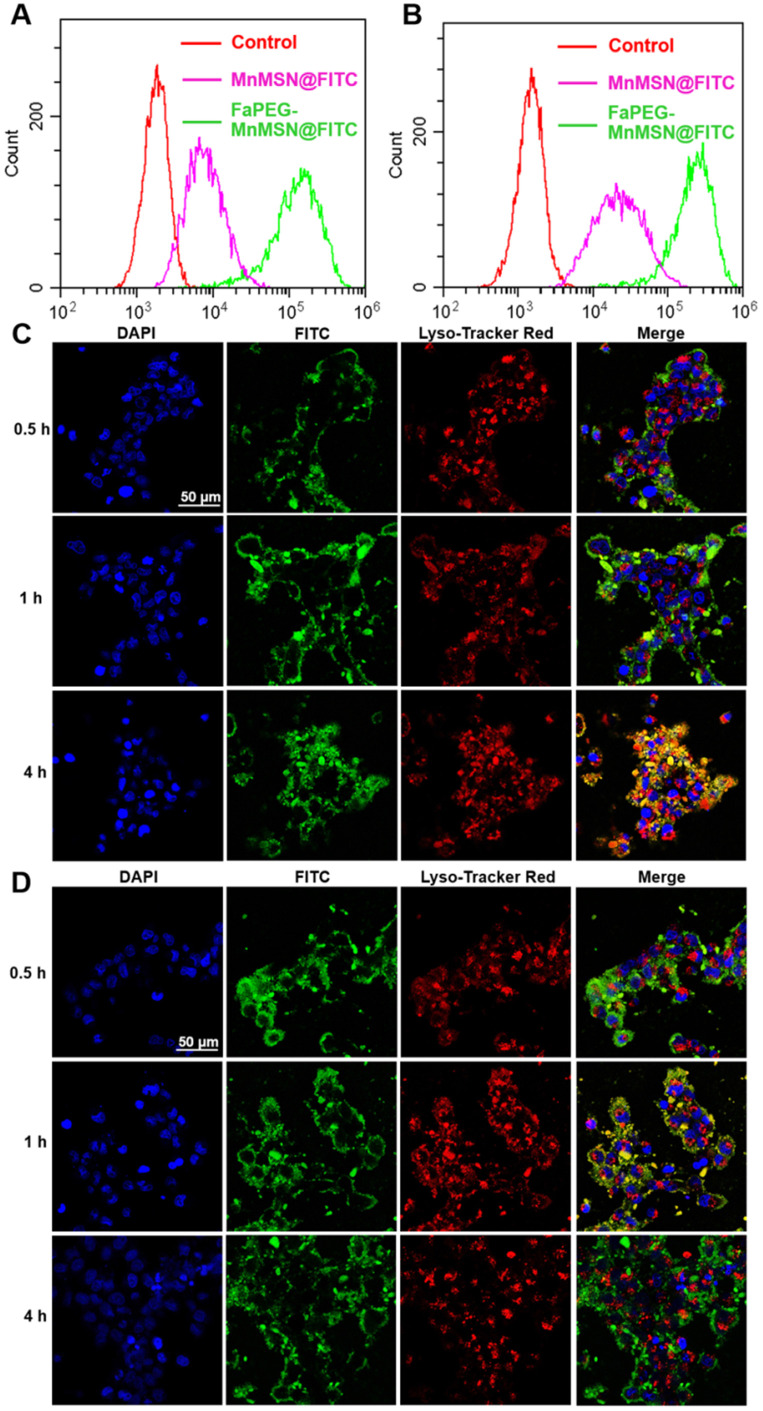
Flow cytometry profiles of HepG2 cells after incubation with MnMSN@FITC or FaPEG-MnMSN@FITC for 1 h (**A**) or 4 h (**B**). Intracellular localization and lysosomal escape observed by CLSM after incubation with MnMSN@FITC (**C**) or FaPEG-MnMSN@FITC (**D**) for 0.5, 1 or 4 h (blue: cell nucleus stained by DAPI; green: FITC labeled MnMSN or FaPEG-MnMSN; red: lysosomes stained by Lyso-Tracker Red; yellow: co-localization areas between lysosomes and MnMSN/FaPEG-MnMSN).

The rapid proliferation of tumor cells leads them to require more folic acid than normal cells. Many studies have confirmed that HepG2 cells have specific endocytic effects on folic acid-modified nanodrugs 41. In our study, the uptake of FaPEG-MnMSN@FITC by HepG2 cells was significantly higher than that of MnMSN@FITC (Figure 4A-B). This feature could facilitate the clearance of GSH by FaPEG-MnMSN@FITC.

Since many antitumor drugs possess the functions of killing tumor cells in the nucleus or cytoplasm, the ability of drugs loaded nanoparticles escaping from lysosomes plays an important role in the determination of their antitumor efficiency. The intracellular localization and lysosomal escape of MnMSN@FITC and FaPEG-MnMSN@FITC were observed by CLSM. In Figure 4C-D, after co-incubation for 0.5 h, FaPEG-MnMSN@FITC exhibited stronger fluorescence signals than that of MnMSN@FITC, indicating that the modification of FaPEG chain could enhance the uptake of nanoparticles by HepG2 cells. 1 h later, yellow fluorescence appeared in Figure 4D, implying that FaPEG-MnMSN@FITC was engulfed by lysosomes. After incubation for 4 h, it was obvious that the overlap between green and red fluorescence disappeared, which meant that FaPEG-MnMSN@FITC escaped from lysosomes. Overall, the above results proved that FaPEG chains could improve the endocytosis efficiency of HepG2 cells, as well as accelerate the escape of nanoparticles from lysosomes.

### GSH concentration, GSH depletion and cytotoxicity

**Figure 5:**
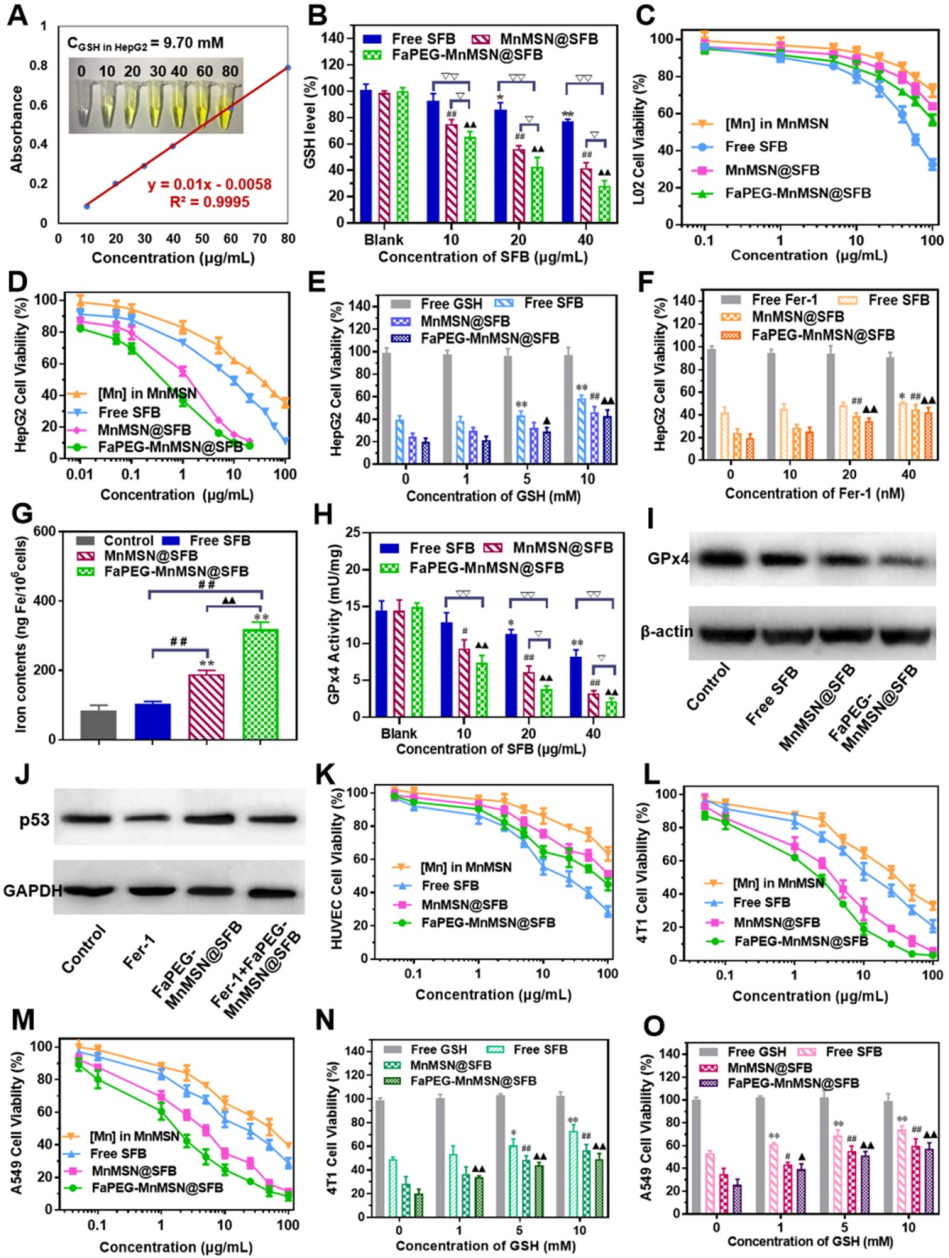
The real GSH concentration in HepG2 cells (**A**). GSH levels of HepG2 cells after incubated with Free SFB, MnMSN@SFB and FaPEG-MnMSN@SFB at concentrations ranging from 0 µg/mL to 40 µg/mL (**B**). *P < 0.05, **P < 0.01, ^#^*P* < 0.05, ^##^*P* < 0.01, ^▲^*P* < 0.05, ^▲▲^*P* < 0.01 vs Blank group. ^▽^*P* < 0.05, ^▽▽^*P* < 0.01 vs cells treated with FaPEG-MnMSN@SFB. Cell viability of L02 (**C**) and HepG2 (**D**) cells after treated with MnMSN, Free SFB, MnMSN@SFB and FaPEG-MnMSN@SFB at different concentrations. The cell viability of HepG2 cells after treated with SFB formulations (containing 20 µg/mL SFB) and GSH (**E**) or Fer-1 (**F**). *P < 0.05, **P < 0.01, ^#^*P* < 0.05, ^##^*P* < 0.01, ^▲^*P* < 0.05, ^▲▲^*P* < 0.01 vs without inhibitor. Iron contents in HepG2 cells after treated with Free SFB, MnMSN@SFB and FaPEG-MnMSN@SFB at the concentration of 20 µg/mL (**G**). **P < 0.01 vs Blank group. ^##^*P* < 0.01 vs cells treated with FaPEG-MnMSN@SFB. ^▲▲^*P* < 0.01 vs cells treated with MnMSN@SFB. GPx4 activity of HepG2 cells after treated with Free SFB, MnMSN@SFB and FaPEG-MnMSN@SFB for different concentrations (**H**). *P < 0.05, **P < 0.01, ^#^*P* < 0.05, ^##^*P* < 0.01, ^▲^*P* < 0.05, ^▲▲^*P* < 0.01 vs Blank group. ^▽^*P* < 0.05, ^▽▽^*P* < 0.01 vs cells treated with FaPEG-MnMSN@SFB. GPx4 expression of HepG2 cells after treated with Free SFB, MnMSN@SFB and FaPEG-MnMSN@SFB at a concentration of 20 µg/mL (**I**). p53 expression of HepG2 cells after treated with Fer-1, FaPEG-MnMSN@SFB and Fer-1+ FaPEG-MnMSN@SFB at a concentration of 20 µg/mL (**J**). Cell viability of HUVEC (**K**), 4T1 (**L**) and A549 (**M**) cells after treated with MnMSN, Free SFB, MnMSN@SFB and FaPEG-MnMSN@SFB at different concentrations. The cell viability of 4T1 (**N**) and A549 (**O**) cells after treated with SFB formulations (containing 20 µg/mL SFB) and GSH. *P < 0.05, **P < 0.01, ^#^*P* < 0.05, ^##^*P* < 0.01, ^▲^*P* < 0.05, ^▲▲^*P* < 0.01 vs without inhibitor.

The real GSH concentration in HepG2 cells is particularly important for experimental design, so we determined the GSH content according to the instructions of reduced glutathione (GSH) colorimetric assay kit. As displayed in Figure 5A, the concentration of GSH in HepG2 cells was 9.70 mM, which was consistent with the high level of GSH (10 mM) we used *in vitro* study.

After swallowed by tumor cells, the GSH-cleaning ability of nanodrugs was the most important issue we wanted to explore. As shown in Figure 5B, the SFB solution exhibited a concentration-dependent ability to consume GSH as SFB can inhibit the function of X_c_^-^ transporters, which transport synthetic material of GSH into cells. As the degradation of MnMSN can deplete the existing GSH in the cells, the intracellular GSH content of the MnMSN@SFB group was markedly lower than that of Free SFB group. Moreover, FaPEG-MnMSN@SFB consumed more GSH than MnMSN@SFB, because HepG2 cells could enhance the uptake of folic acid-modified nanodrugs.

Consumption of GSH will cause redox imbalance and death of tumor cells. As displayed in Figure 5D, both MnMSN and Free SFB exhibited concentration-dependent cytotoxicity to HepG2 cells with IC_50_ of 31.44 ± 2.75 µg/mL (Mn) and 7.58 ± 1.18 µg/mL (SFB), respectively. The IC_50_ value of MnMSN@SFB was 1.13 ± 0.32 µg/mL (SFB), which was significantly smaller than that of Free SFB treatment group. This was because MnMSN@SFB inhibited the synthesis of GSH and increased the consumption of GSH, which eliminated intracellular GSH and exhibited potent cytotoxicity. For tumor-specific uptake, the IC_50_ of FaPEG-MnMSN@SFB was further reduced compared to that of MnMSN@SFB. As for normal liver cells (L02 cells), for the intracellular GSH content is low, MnMSN cannot be quickly degraded and exhibited low cytotoxicity (Figure 5C). In particular, free SFB group possessed higher cytotoxicity than MnMSN@SFB and FaPEG-MnMSN@SFB. This is due to the low content of GSH in normal cells which cannot trigger the degradation of MnMSN and release the drug. This indicated that MnMSN@SFB and FaPEG-MnMSN@SFB were safe for normal cells.

To corroborate the mechanisms of “GSH starvation” therapy, HUVEC, 4T1 and A549 cells were selected to carry out the cytotoxicity experiments. In Figure 5K-M, the cytotoxicity of SFB formulations against 4T1 and A549 cells was significantly higher than HUVEC cells, which could be ascribed to the high level of GSH in tumor cells compared to that of normal cells. Afterwards, the cytotoxicity of SFB-loaded nanodrugs was further investigated through the addition of GSH (0 ~ 10 mM). As exhibited in Figure 5N-O, with the increase of GSH concentration, the toxicity of SFB nanoparticles decreased gradually, indicating that the depletion of intracellular GSH was the main cause of triggering cell death.

### Mechanism of GSH-nanocleaner induced cell death via ferroptosis and apoptosis

Recently, ferroptosis has aroused extensive attention in the medical community due to its effective lethality against tumor cells. It is a new form of regulated cell death caused by PL-PUFA-OOH accumulation (oxidized by ROS), which will result in redox dysregulation and rupture of the cell membrane 42, 43. The GSH-dependent GPx4 could reduce toxic PL-PUFA-OOH to non-toxic PL-PUFA-OH 8, thereby reducing the accumulation of lipid free radicals to prevent the occurrence and progression of ferroptosis (Figure 1B). In addition, the activation of GPx4 is highly dependent on the intracellular GSH. Based on above cognitions, the exhaustion of GSH will inhibit GPx4 activity of tumor cells, and thus exert a ferroptosis-inducing effect. As shown in Figure 5H-I, Free SFB could significantly inhibit the activity and expression of GPx4 at concentrations higher than 20 μg/mL. FaPEG-MnMSN@SFB demonstrated the strongest effect on inhibiting the activity of GPx4 among the three formulations at concentrations higher than 10 µg/mL. The results of this study correspond to the results of intracellular GSH determination (Figure 5B).

As displayed in Figure 5E, the cytotoxicity of Free SFB was markedly decreased (*P* < 0.01) when GSH concentration was higher than 5 mM. Because external GSH could antagonize the inhibition of GSH biosynthesis induced by SFB. As for MnMSN@SFB and FaPEG-MnMSN@SFB, upon the addition of GSH, the release of SFB was advanced owing to the decomposition of MnMSN. Meanwhile, the consumption of intracellular GSH caused by MnMSN was reduced with the increase of external GSH. Therefore, the more exogenous GSH was added, the more the cytotoxicity of two SFB formulations (MnMSN@SFB and FaPEG-MnMSN@SFB) approached that of Free SFB. Fer-1, as the inhibitor of ferroptosis, could significantly alleviate the cytotoxicity of MnMSN@SFB and FaPEG-MnMSN@SFB (*P* < 0.01, Figure 5F) when the concentration of Fer-1 was more than 20 nM.

Iron participates in many life activities, such as transport of oxygen, synthesis of hemoglobin and synthesis of DNA. Besides, intracellular iron is indispensable for ferroptosis, the iron content increases markedly during ferroptosis 44, 45. To verify the occurrence of ferroptosis, the iron content of HepG2 cells were determined after treated with SFB nanodrugs for 24 h. Iron content was significantly increased in MnMSN@SFB and FaPEG-MnMSN groups compared to Control and Free SFB groups, indicating that SFB formulations could induce the ferroptosis of HepG2 cells to a large extent (Figure 5G).

Although p53-mediated apoptosis, senescence and cell-cycle arrest are supposed to be critical obstacles against cancer development 46, increasing evidences indicate that metabolic activities of p53 are also crucial. Researchers found that p53 could inhibit the uptake of cystine by down-regulating the expression of SLC7A11, a key ingredient of Xc^-^ transport system, thereby inhibiting the activity of GPx4 and enhancing the sensitivity of cells to ferroptosis 47. FaPEG-MnMSN@SFB treatment caused the upregulation of p53 protein in HepG2 cells, which was in accordance with a possible effect in apoptosis and ferroptosis regulations (Figure 5J). Moreover, the addition of Fer-1 could repress the expression of p53 protein by inhibiting the ferroptosis of SFB formulation (Figure 5J).

To further clarify the mechanism of ferroptosis induced by nanocleaner, intracellular ROS, PL-PUFA-OOH and cell membrane morphology were investigated upon various treatments, separately. First of all, different types of ROS were assessed by staining •O_2_^-^, •OH and ROS with DHE, HPF and DCFH-DA. FaPEG-MnMSN@SFB treatment could cause GSH depletion by consumption of intracellular GSH and inhibition of GSH biosynthesis. Since GSH depletion would lead to more H_2_O_2_ remained in HepG2 cells 28, 29, •OH produced by Fenton-like reaction of H_2_O_2_ under the catalysis of Mn^2+^/Fe^2+^ also increased, as evidenced by the strongest fluorescence among three groups (Figure 7B). In addition, •O_2_^-^ could reduce Fe^3+^ to Fe^2+^ in Fe-S centers of proteins, resulting in the inactivation of enzymes, which in turn fueled Fenton chemistry 48. SFB, an inhibitor of Xc^-^ transporter inhibitor, could block the biosynthesis of GSH, leading to the accumulation of intracellular ROS. Thus, with the increase of SFB concentration, the fluorescence intensity of ROS increased (Figure 7A-C). The produced ROS would further oxidize PL-PUFA-OH to PL-PUFA-OOH. As demonstrated in Figure 7D, with the increase of SFB concentration, the fluorescence intensity of PL-PUFA-OOH was enhanced. After combining with MnMSN, the fluorescence intensity of cells was further enhanced. These results indicated that the strategies of GSH exhaustion and GSH synthesis inhibition could achieve a synergistic ferroptosis-inducing effect. Furthermore, GSH-nano cleaner exhibited the most PL-PUFA-OOH accumulation among three experimental groups, which could be attributed to the modification of FaPEG chains (Figure 7D). With the accumulation of PL-PUFA-OOH, the cells will eventually shift towards ferroptosis. As shown in Figure 7E, after treatment with 10 µg/mL of Free SFB, HepG2 cell membranes remained intact. With the increase of SFB concentration, the HepG2 cell membranes lost their integrity and the fluorescence signal became weak. This was because the accumulation of PL-PUFA-OOH caused the rupture of cell membranes. Compared to other groups, GSH-cleaner exerted the most destructive effect on cell membranes. When the drug concentration was 20 µg/mL, almost all cell membranes were disassembled. And fluorescence signal of cell membranes nearly disappeared at the SFB concentration of 40 µg/mL.

**Figure 6:**
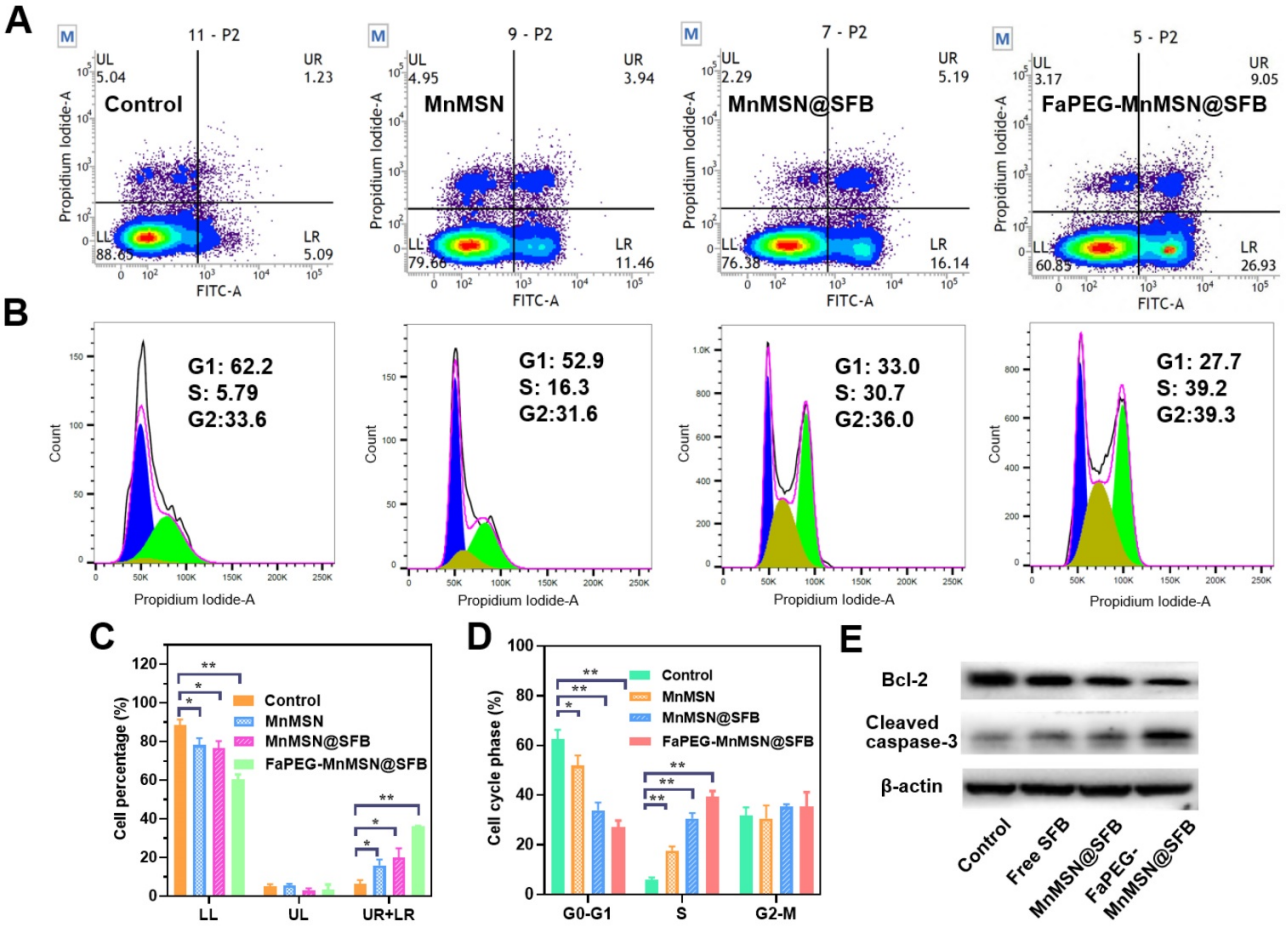
Flow cytometric analyzed Control, Free SFB, MnMSN@SFB and FaPEG-MnMSN@SFB ([SFB]=20 µg/mL) on the apoptosis of HepG2 cells using the Annexin V-FITC/PI (**A**). HepG2 cells cycle perturbations induced by Control, Free SFB, MnMSN@SFB and FaPEG-MnMSN@SFB at a concentration of 20 µg/mL (**B**). Cell apoptosis percentage (**C**) and cell cycle phase (**D**) induced by Control, Free SFB, MnMSN@SFB and FaPEG-MnMSN@SFB ([SFB]=20 µg/mL). **P < 0.01, *P < 0.05 vs Control group. Bcl-2 and Cleaved caspase-3 expression of HepG2 cells after treated with Control, Free SFB, MnMSN@SFB or FaPEG-MnMSN@SFB (**E**).

**Figure 7:**
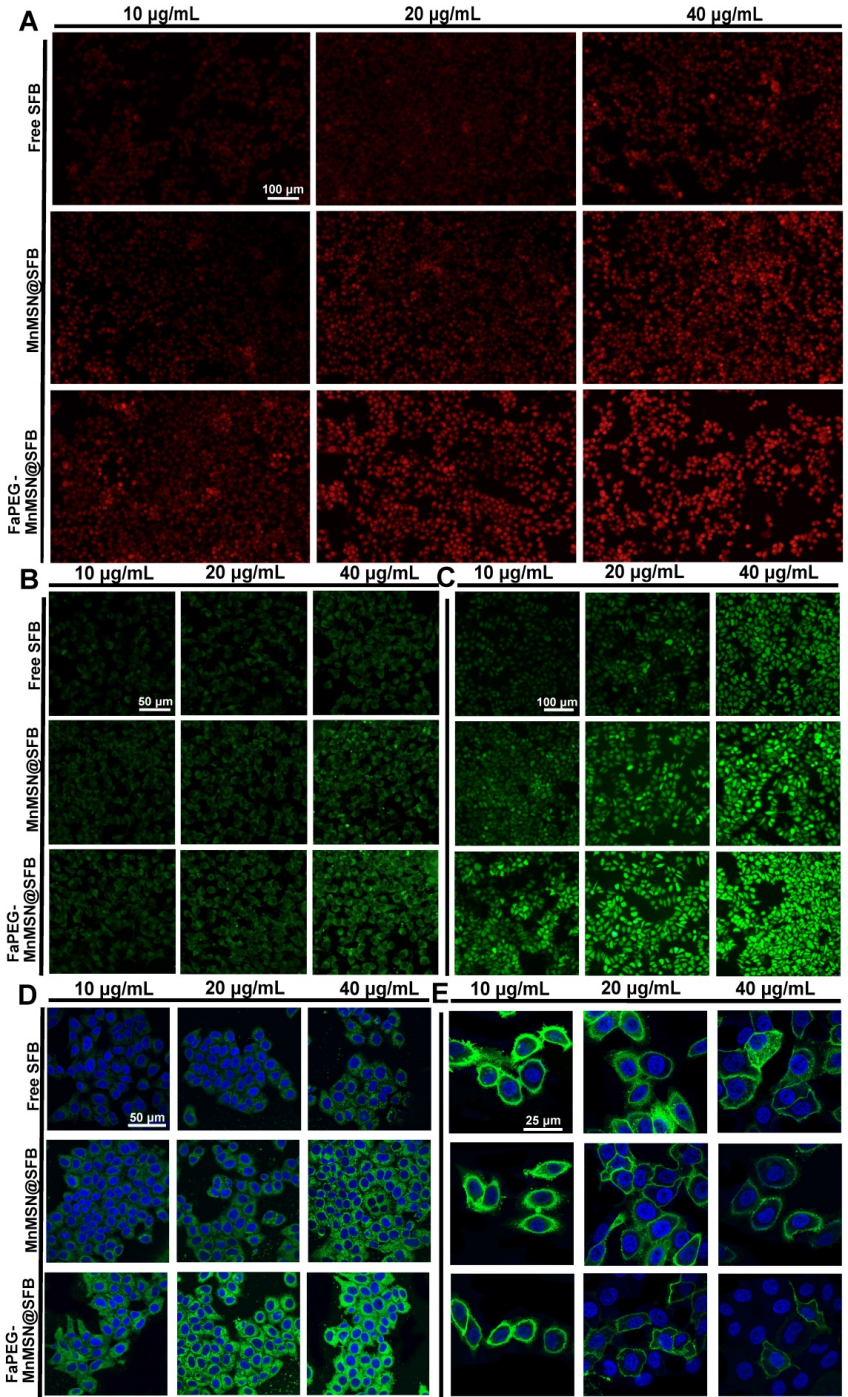
Superoxide anion (•O_2_^-^) (**A**), hydroxyl radical (•OH) (**B**) and ROS (**C**) detection assays of HepG2 cells stained with DHE, HPF and DCFH-DA after incubated with Free SFB, MnMSN@SFB or FaPEG-MnMSN@SFB at concentrations ranging from 10 µg/mL to 40 µg/mL. PL-PUFA-OOH (**D**) and membrane morphological changes (**E**) of HepG2 cells stained with C11-BODIPY^581/591^ and DIO after incubated with Free SFB, MnMSN@SFB or FaPEG-MnMSN@SFB at concentrations ranging from 10 µg/mL to 40 µg/mL.

Mitochondria, the main sites for aerobic respiration and ROS generation, require a certain amount of GSH to maintain the redox balance. However, owing to the lack of glutathione synthetase, mitochondria cannot synthesize GSH autonomously, but they can obtain GSH from the cytoplasm 49. The depletion of cytoplasmic GSH will affect the GSH content and functions of mitochondria. The loss of intracellular GSH occurs prior to the destruction of mitochondria, the release of Cyt c and the activation of caspase, which is considered as an early event in the process of cell apoptosis in response to various stimulations 50-53. In addition, the -SH group in GSH is crucial for maintaining the status of DNA repair and expression in the nucleus, and the inhibition of DHA synthesis and repair will promote the apoptosis of tumor cells. As presented in Figure 6A and 6C, compared with the Control group (6.12 ± 2.63%), the proportion of early apoptotic and late apoptotic cells were significantly increased in the Free SFB-treated group (16.04 ± 3.02%). This result indicated that the consumption of intracellular GSH could promote apoptosis to some extent for SFB could inhibit the synthesis of GSH. Furthermore, GSH-cleaner possessed the largest proportion of early and late apoptosis (36.05 ± 0.20%), which may be caused by dual GSH depletion.

The rapid depletion of GSH in cells causes damage to mitochondria which would cause changes in apoptosis-related factors such as Bcl-2, and eventually caused caspase activation. In Figure 6E, compared to the Control group, the expression of Bcl-2 was reduced in the Free SFB-treated group, while the expression of cleaved caspase-3 was increased. Similar to the results of inducing ferroptosis, FaPEG-MnMSN@SFB had the efficient GSH clearance ability, so it could significantly inhibit the expression of Bcl-2 and increase the expression of cleaved caspase-3, thus exerting the strongest effect of inducing apoptosis (36.05 ± 0.20%).

Although the concentration of GSH in nucleus is very low, it has been proven that the nuclear GSH is vital in cell cycle 53-55. In our research, the clearance of GSH greatly affected the cell proliferation cycle. As displayed in Figure 6B and 6D, after treatment with Free SFB, the S phase of tumor cells was significantly longer (*P* < 0.01) than that of the Control group. This was because the absence of GSH affected the synthesis of DNA 3-5. Overall, FaPEG-MnMSN@SFB exhibited the longest S phase arrest (*P* < 0.01) owing to the dual GSH depletion.

### Pharmacokinetic study

**Figure 8:**
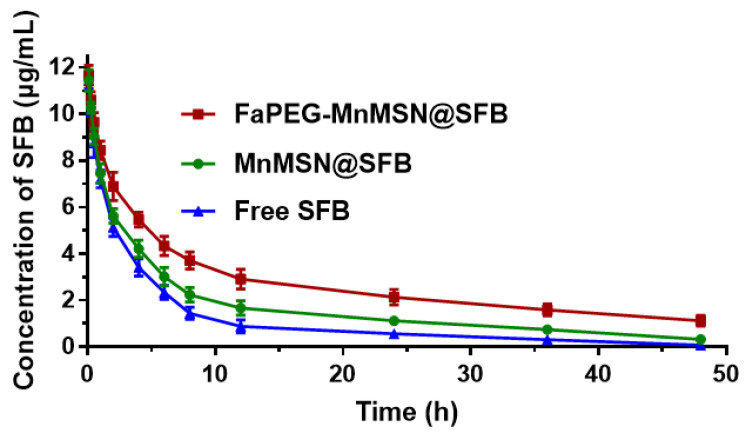
The time-concentration profiles of SFB in SD rats after intravenous injection (n=6).

In this study, SFB was loaded into FaPEG-MnMSN with pH/GSH-triggered release, long circulation and targeted delivery properties. Then, its PK feature in plasma was investigated. The time-concentration profiles of SFB formulations were drawn in Figure 8 and the main PK parameters were calculated in Table 1. By comparison with Free SFB group, controlled release properties of MnMSN@SFB and FaPEG-MnMSN@SFB were proved by markedly prolonged t_1/2_ (P < 0.01), increased AUC_0-t_ (P < 0.01) and reduced clearance (CL) (P < 0.01). Moreover, compared to MnMSN@SFB, the prolonged t_1/2_ (P < 0.01), improved AUC_0-t_ (P < 0.01) and prolonged MRT (P < 0.01) of FaPEG-MnMSN@SFB could be attributed to increased circulation time and reduced drug leakage of PEG modification. Afterwards, the modification of PEG would form nonspecific steric hindrance between nanoparticles to prevent the combination of serum proteins with nanoparticles, thereby reducing uptake by reticuloendothelial system 34-36.

### *In vivo* fluorescence imaging and MRI imaging studies

**Figure 9:**
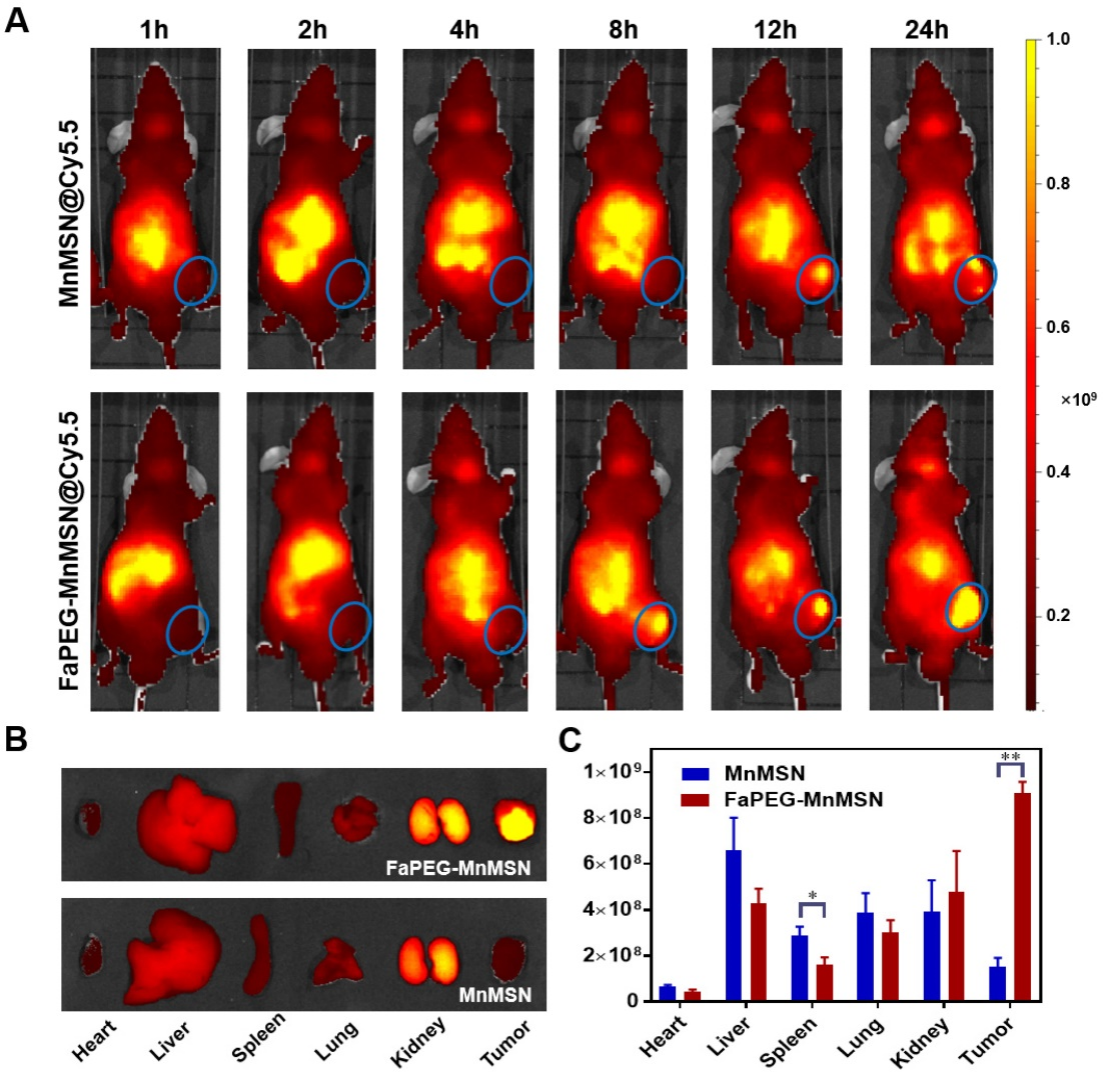
Whole-body fluorescence images of HepG2 tumor-bearing nude mice after injection of Cy5.5-labeled MnMSN or Cy5.5-labeled FaPEG-MnMSN (n=5 in each group) (**A**) (the HepG2 tumor was marked by blue ellipse). The fluorescence images (**B**) and intensities (**C**) of major organs and tumors in HepG2 tumor-bearing nude mice after injection for 24 h. **P < 0.01, *P <0.05 vs MnMSN group.

To investigate the tumor-targeting effect of designed nanoparticles, Cy5.5 was employed to label MnMSN and FaPEG-MnMSN for *in vivo* fluorescence imaging. In Cy5.5 labeled FaPEG-MnMSN group, the fluorescence signal at tumor site appeared at 8 h after injection, and gradually increased from 8 to 24 h, while the fluorescence in the liver gradually weakened. By comparison, the appearance of the fluorescence of Cy5.5 labeled MnMSN appeared at tumor site occurred a little later (approximately 12 h), and then gradually disappeared. The difference between the two groups was mainly caused by long blood circulation of PEG chain and active targeting of Fa. It could be seen from Figure 9B-C that the Cy5.5 labeled FaPEG-MnMSN was mainly enriched in tumor and kidney, while the Cy5.5 labeled MnMSN was mainly concentrated in kidney and liver. This result not only showed that FaPEG chains could enhance the uptake of nanoparticles by tumor tissue, but also proved that MnMSN or FaPEG-MnMSN could be degraded *in vivo* and metabolized by kidney.

**Figure 10:**
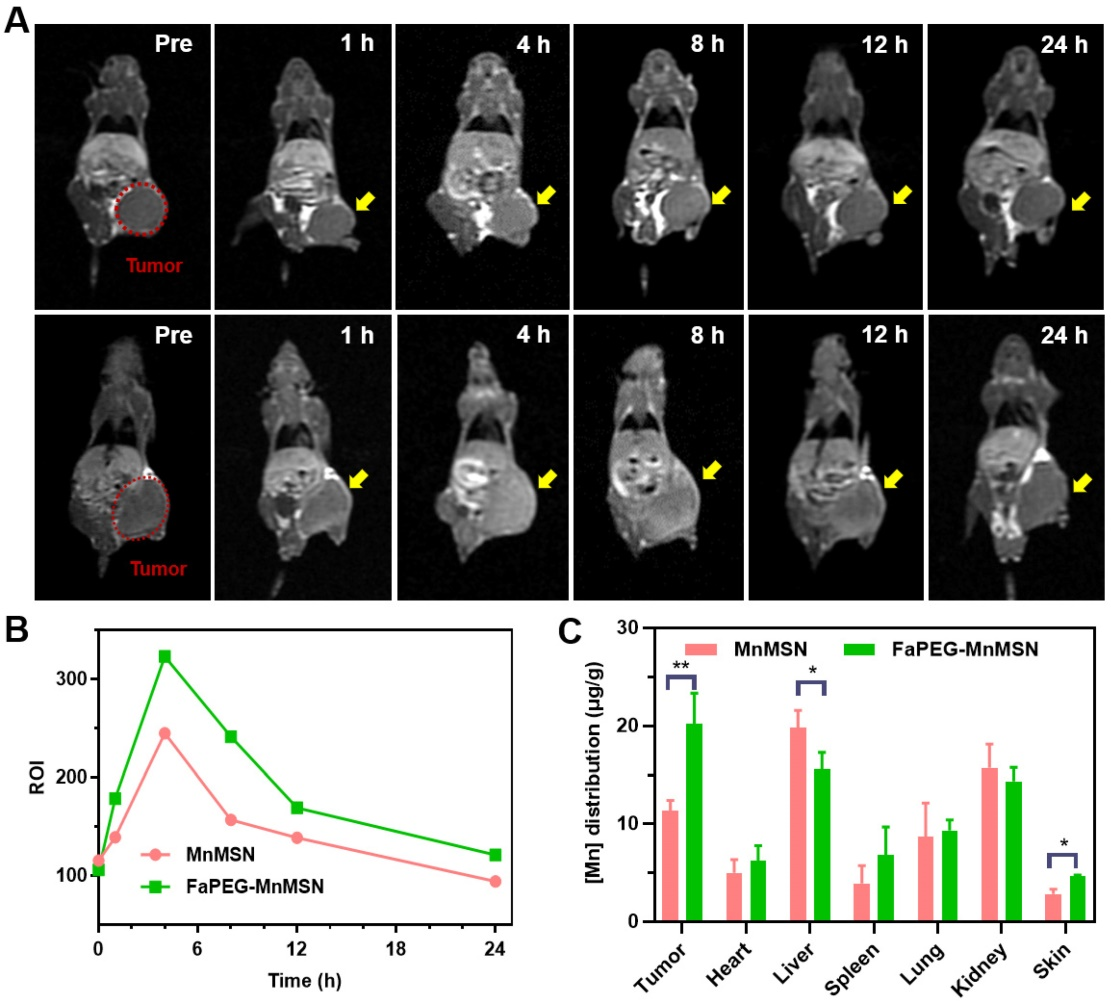
*In vivo* MRI of HepG2 tumor-bearing nude mice after injection of MnMSN or FaPEG-MnMSN during 24 h (1.5 mg Mn/kg) (n=3 in each group) (**A**) (tumor was marked in Pre Figure). T1-MRI signal intensities of tumor tissues after injection of MnMSN or FaPEG-MnMSN for varied durations (**B**). *In vivo* biodistributions of Mn element in 4 h after injection (**C**). **P < 0.01, *P < 0.05 vs MnMSN group.

After injection of MnMSN or FaPEG-MnMSN, the MRI signals of tumor sites in tumor bearing nude mice showed trends of enhancement (Figure 10A). In FaPEG-MnMSN group, at 1 h post injection, the MRI signal of tumor site was significantly enhanced, which proved that FaPEG-MnMSN could quickly reach the tumor site, and then released Mn^2+^ reduced by high concentration of GSH. MRI signals of FaPEG-MnMSN peaked at 4 h and then decreased progressively. In contrast, the MRI signal of tumor site in MnMSN group was lower than that of FaPEG-MnMSN group (Figure 10B). In addition, the enhanced MRI signal could accelerate the clinical diagnosis of tumors. After that, ICP-MS was used to measure the Mn content of tumors and major organs. As displayed in Figure 10C, the concentration of Mn^2+^ at tumor site of FaPEG-MnMSN was markedly higher than that of MnMSN group (*P* < 0.05). It is worth noting that although the fluorescence signal (Figure 9A-B) and Mn element (Figure 10C) were abundant in the liver, the MRI signal did not increase significantly, which was due to the comparably lower content of GSH in liver cells than that of tumor cells, as such, few nanocarriers could degrade and release Mn^2+^ in the liver. Comparison of the two imaging methods showed that FaPEG-MnMSN could degrade specifically in tumor tissues, while being relatively safe for normal tissues.

### *In vivo* anti-tumor efficiency of the designed nanocleaner

**Figure 11:**
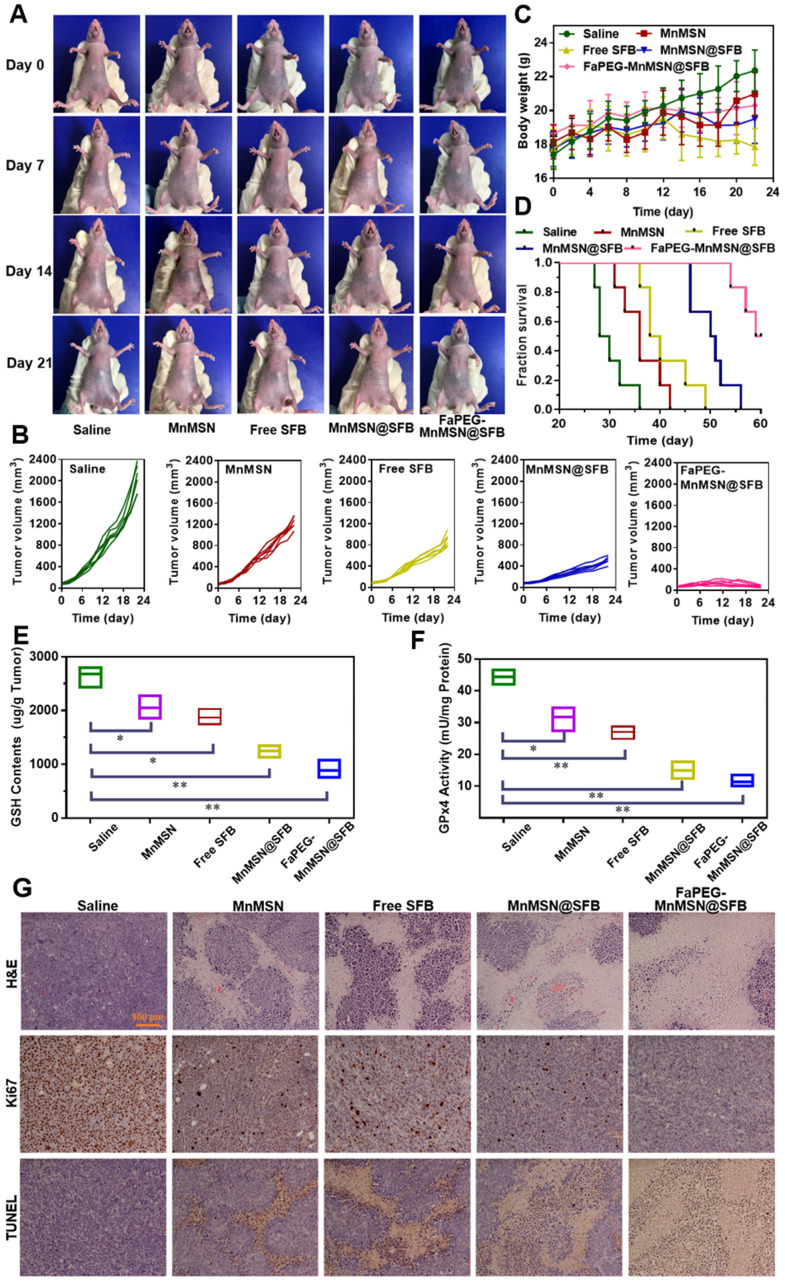
Typical images of HepG2 tumor-bearing mice at predetermined time intervals after injection of Saline, MnMSN, Free SFB, MnMSN@SFB, or FaPEG-MnMSN@SFB at a SFB-equivalent dose of 10 mg/kg (**A**). The tumor volume changes (**B**), body weight changes (**C**) and Kaplan-Meier survival curves (**D**) of nude mice in each group during the treatment period (n=6). The intratumoral GSH (**E**) and GPx4 (**F**) levels after various treatment. The H&E, Ki67 and TUNEL staining images of tumor tissues indicating the tissue necrosis, cell proliferation and cell apoptosis (**G**), scale bar is 100 µm.

Based on the excellent inhibitory effect of FaPEG-MnMSN@SFB on HepG2 cells *in vitro*, the *in vivo* therapeutic efficacy of the above nano "GSH-cleaner" was evaluated in HepG2 tumor-bearing mice. Figure 11A presented the survival state of mice during the treatment period. On day 21, the tumors in FaPEG-MnMSN@SFB group almost disappeared, revealing the best anti-tumor efficacy. After 22 days of treatment, the average tumor volumes presented in Figure 11B were 2060.99 ± 259.55 mm^3^, 1243.58 ± 115.47 mm^3^, 894.25 ± 121.67 mm^3^, 515.23 ± 73.46 mm^3^, and 70.50 ± 18.56 mm^3^ in the group of Saline, MnMSN, Free SFB, MnMSN@SFB, and FaPEG-MnMSN@SFB, respectively. Compared with the other four groups, FaPEG-MnMSN@SFB treated group exhibited a marked reduction in tumor growth, which may have been dependent on the active targeting and long-term circulation associated with the FaPEG modification. In Figure 11C, the body weight in the Saline group displayed a distinct increase during the treatment, which may be caused by the rapid growth of tumor. By contrast, the body weight of Free SFB treated group presented slight loss from day 12, indicating that the mice in Free SFB group had a poor life quality, which may have been due to a high toxicity to primary organs caused by the systemic distribution of SFB. The median survival time was 29, 36, 39, 50.5, and 64.5 days in Saline, MnMSN, Free SFB, MnMSN@SFB, and FaPEG-MnMSN@SFB groups, respectively (Figure 11D). Overall, GSH-cleaner treated group possessed the longest median survival time, which was attributed to the best therapeutic efficacy among all groups. In addition, the GSH content and GPx4 activity were decreased markedly after treatment with GSH-cleaner among all groups, demonstrating the redox homeostasis was destroyed (Figure 11E-F).

**Figure 12:**
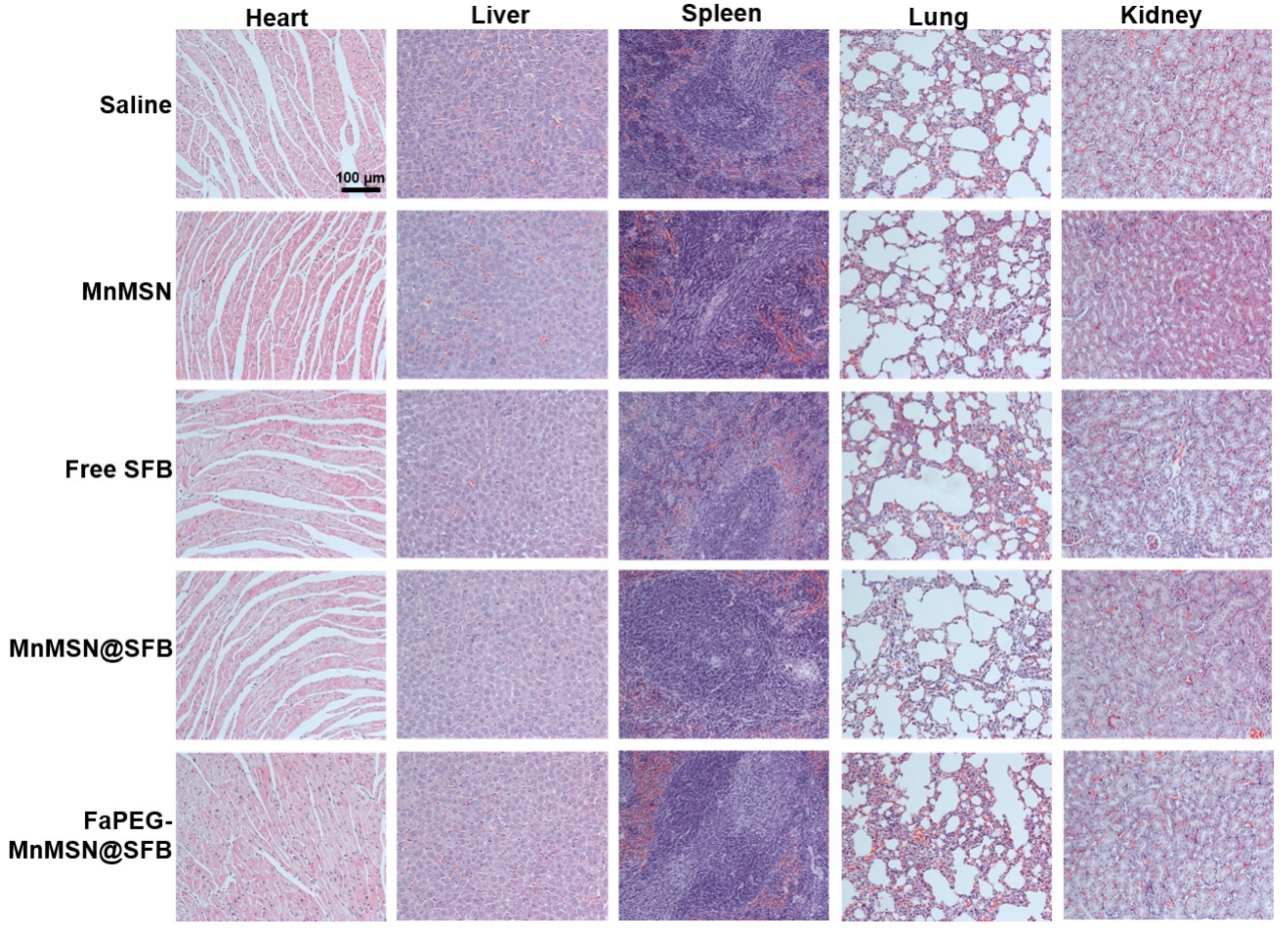
*In vivo* toxicity evaluations of saline, Free SFB and SFB formulations. H&E staining images of normal organs in each group, the scale bar is 100 µm.

To further assess the anticancer efficiency of SFB-loaded GSH-cleaner, major organs were collected for histopathological analysis. As shown in Figure 11G, results of H&E staining suggested that FaPEG-MnMSN@SFB treated group displayed the largest area of tumor tissue necrosis among groups. Moreover, the Ki67 and TUNEL staining results proved that the least tumor cell proliferation and the most tumor cell apoptosis in FaPEG-MnMSN@SFB treated group. Besides, H&E staining results of major organs showed that FaPEG-MnMSN@SFB did not result in noticeable damage to the major organs (Figure 12). This was in accordance with the *in vitro* cytological result that FaPEG-MnMSN@SFB substantially decreased the toxicity to L02 cells.

### Biosafety of nanocleaner

Nowadays, an increasing number of scholars paid their attention to biodegradability and metabolizability of nanomaterials, which play a considerable role in *in vivo* biosafety and clinical transformation. According to the above experimental results, the biodegradability of FaPEG-MnMSN was proved by the Mn ion degraded curve (Figure 3C), Si ion degraded curve (Figure 3D) and TEM images (Figure 3E-F). The metabolizability of FaPEG-MnMSN was confirmed by the fluorescence distribution in major organs (Figure 9A-C) and MRI images (Figure 10A). At 24 h, the fluorescence intensity of kidney was second only to tumor in FaPEG-MnMSN group, revealing that the designed nanocleaner could be metabolized by kidney. To further evaluate the hemocompatibility of nanocleaner, hemolysis test was conducted. As displayed in Figure S12, the hemolysis rate of MnMSN reached 27.85 ± 4.85% while modified FaPEG-MnMSN reduced to 2.34 ± 0.53% at 2 mg/mL, indicating that the modification of FaPEG could significantly reduce the hemolytic toxicity and improve the hemocompatibility of MnMSN. In conclusion, the designed nanocleaner with excellent biosafety is expected to be used for the delivery of anti-tumor drugs.

## Conclusions

In general, targeted SFB-loaded manganese doped silica nanocleaner was successfully constructed for cancer GSH activatable cascade reaction to realize collaborative cancer therapy. The depletion of GSH (caused by the decomposition of MnMSN) and inhibition of GSH biosynthesis (caused by SFB) played a synergistic role in inducing apoptosis and ferroptosis. Intracellular GSH would control the •OH production by regulating Mn^2+^ degraded from FaPEG-MnMSN, so as to avoid the side effects by excessive •OH. GSH-activated MRI signals could be used for accurate tumor diagnosis, as well as for monitoring the antitumor effect of tumor site. With the further assistance of tumor-targeting and long-circulation of FaPEG, the synthesized GSH-cleaner exhibited a comprehensive anti-tumor efficacy. Over all, this study presented a multi-angle “GSH-starvation” strategy, which was expected to be a candidate for the next generation of cancer treatment.

## Supplementary Material

Supplementary figures.

## Figures and Tables

**Table 1:** Main pharmacokinetic parameters of SFB after vein injection in SD rats (n=6)

Parameters	Free SFB	MnMSN@SFB	FaPEG-MnMSN@SFB
t_1/2_ (h)	8.95±2.73	14.99±2.26**	25.53±2.57**^##^
AUC_0-t_ (µg/mL*h)	54.35±4.58	80.40±7.81**	129.98±14.65**^##^
V_ss_ (mg/kg)/(µg/mL)	1.91±0.23	2.02±0.23	2.09±0.27
CL (L/kg/h)	0.18±0.01	0.12±0.01**	0.06±0.01**^##^
MRT (h)	10.51±0.64	17.57±2.20**	32.74±3.74**^##^

*P < 0.05, **P < 0.01 vs Free SFB group; ^#^*P* < 0.05, ^##^*P* < 0.01 vs MnMSN@SFB group.
