# Supplementary material for: Targeted Manganese doped silica nano GSH-cleaner for treatment of Liver Cancer by destroying the intracellular redox homeostasis
Source: Theranostics. 2020 Aug 2;10(21):9865–87. doi: 10.7150/thno.46771 (PMC7449918; doi:10.7150/thno.46771)
Supplement: Supplementary file 1 — Supplementary figures. [file thnov10p9865s1.pdf]

Supplementary materials

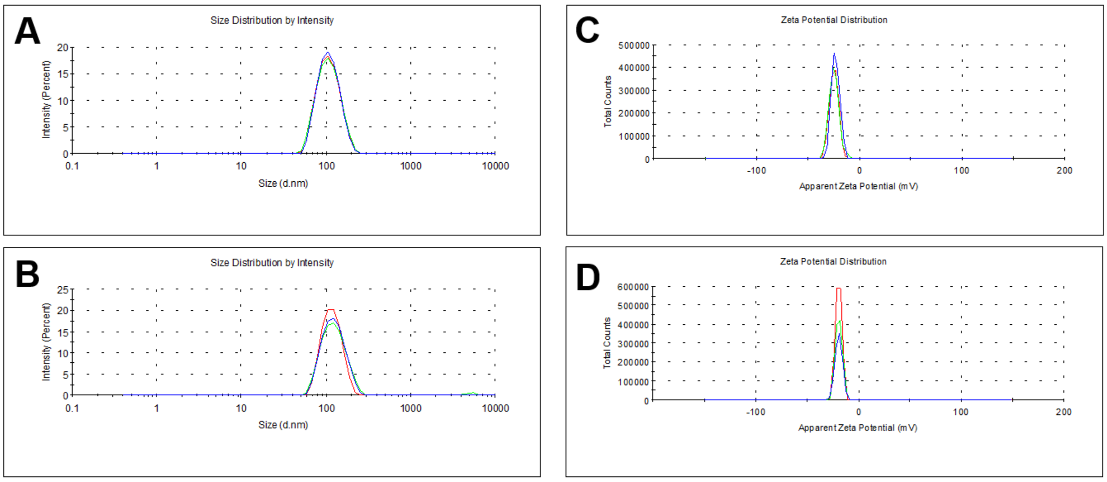

Figure S1. Particle sizes of MnMSN (A) and FaPEG-MnMSN (B). Zeta potentials of MnMSN (C) and FaPEG-MnMSN (D).

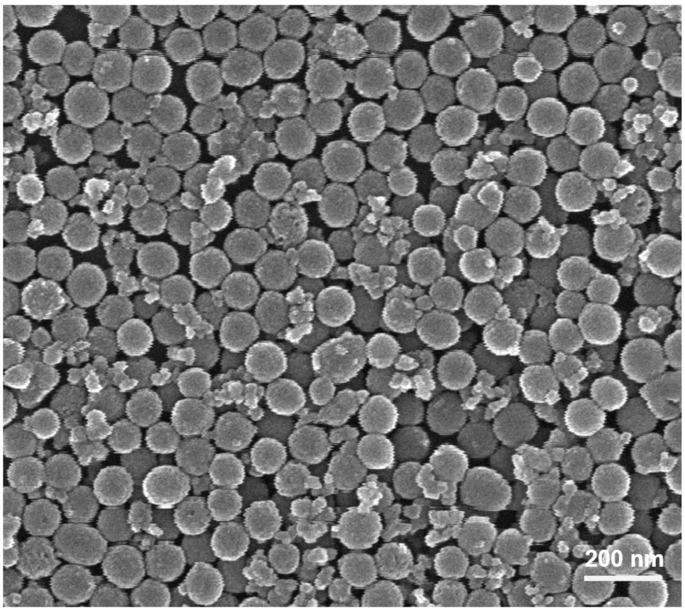

Figure S2. SEM image of FaPEG-MnMSN.

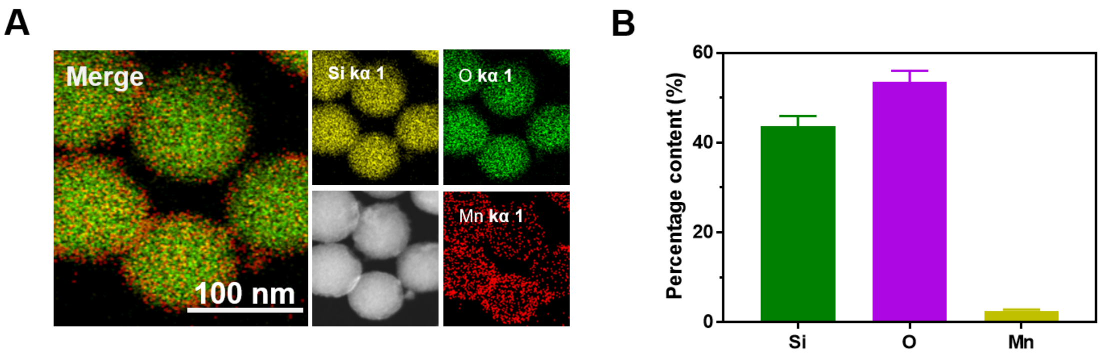

Figure S3. Element mappings (A) and Percentage content (B) of Si, O, and Mn in MnMSN.

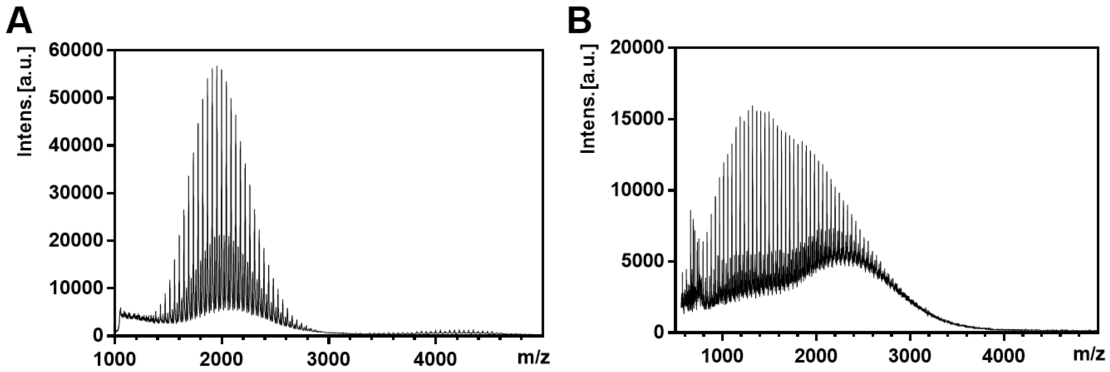

Figure S4. MOLDI-TOF-MS spectrum of mPEG-saline (A) and FaPEG-saline (B).

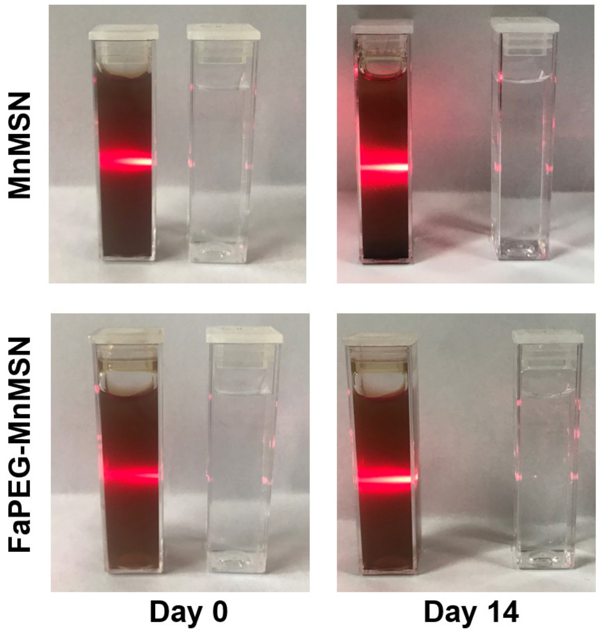

Figure S5. The Tyndall phenomenon of MnMSN and FaPEG-MnMSN during 14 days.

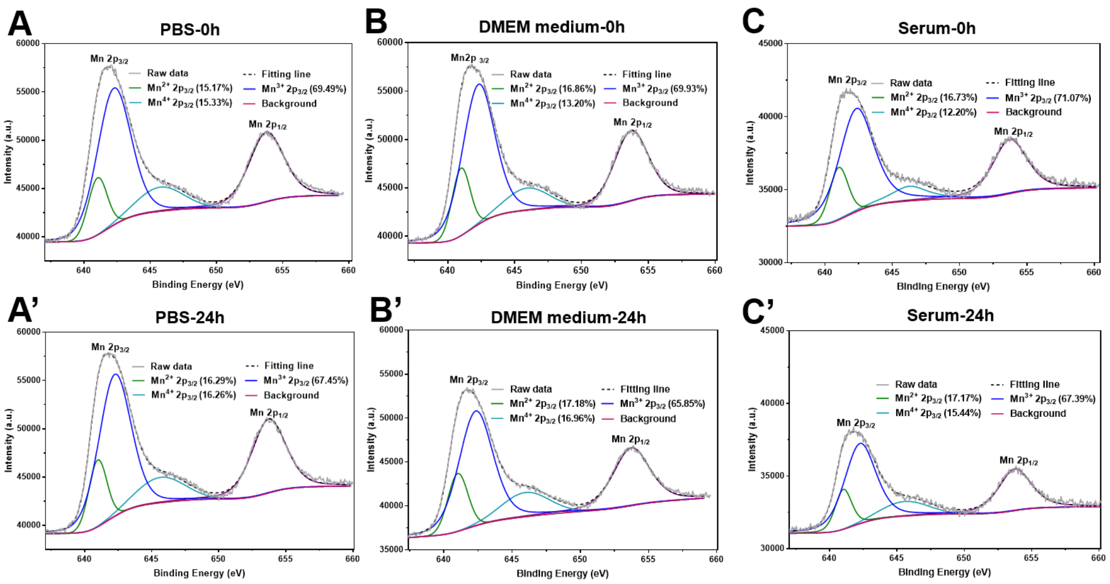

Figure S6. The valence changes of Mn ions in FaPEG-MnMSN at various physiological conditions during 24 h.

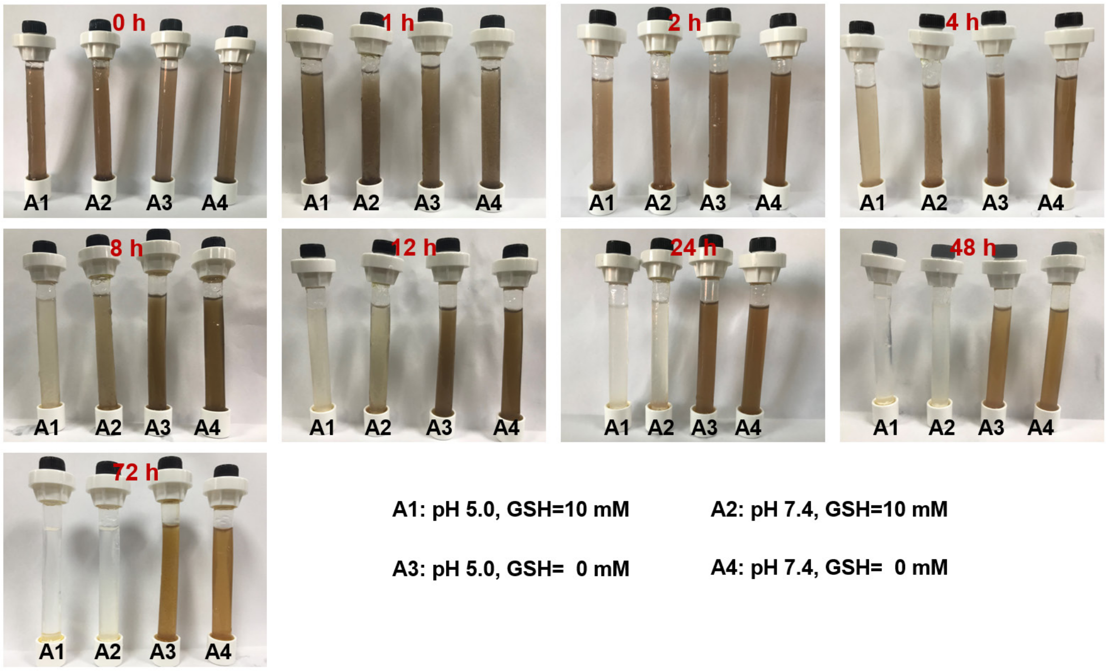

Figure S7. Color changes of FaPEG-MnMSN dispersion during the degradation under different GSH and pH conditions.

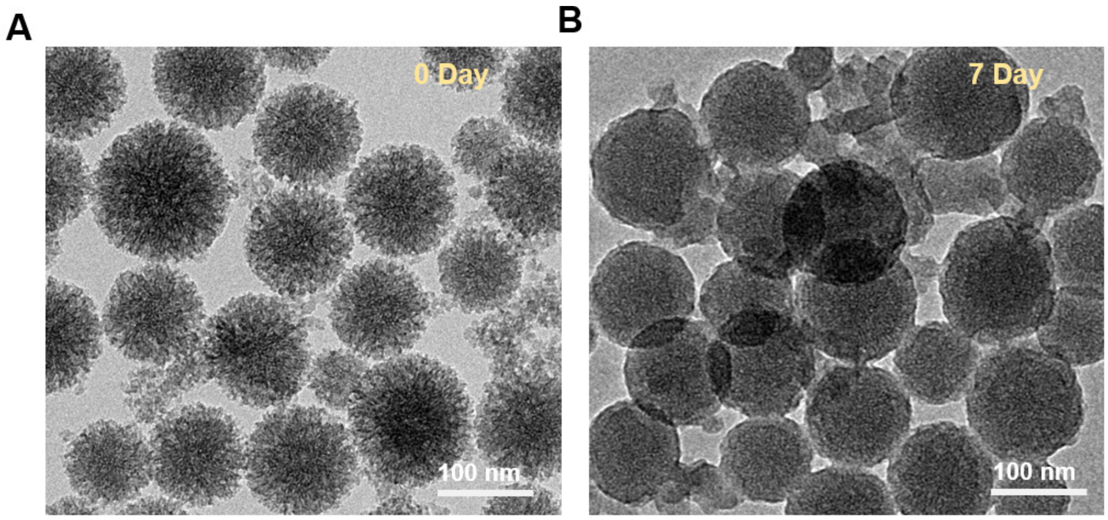

Figure S8. The degradation of FaPEG-MnMSN for 7 days under physiological environment.

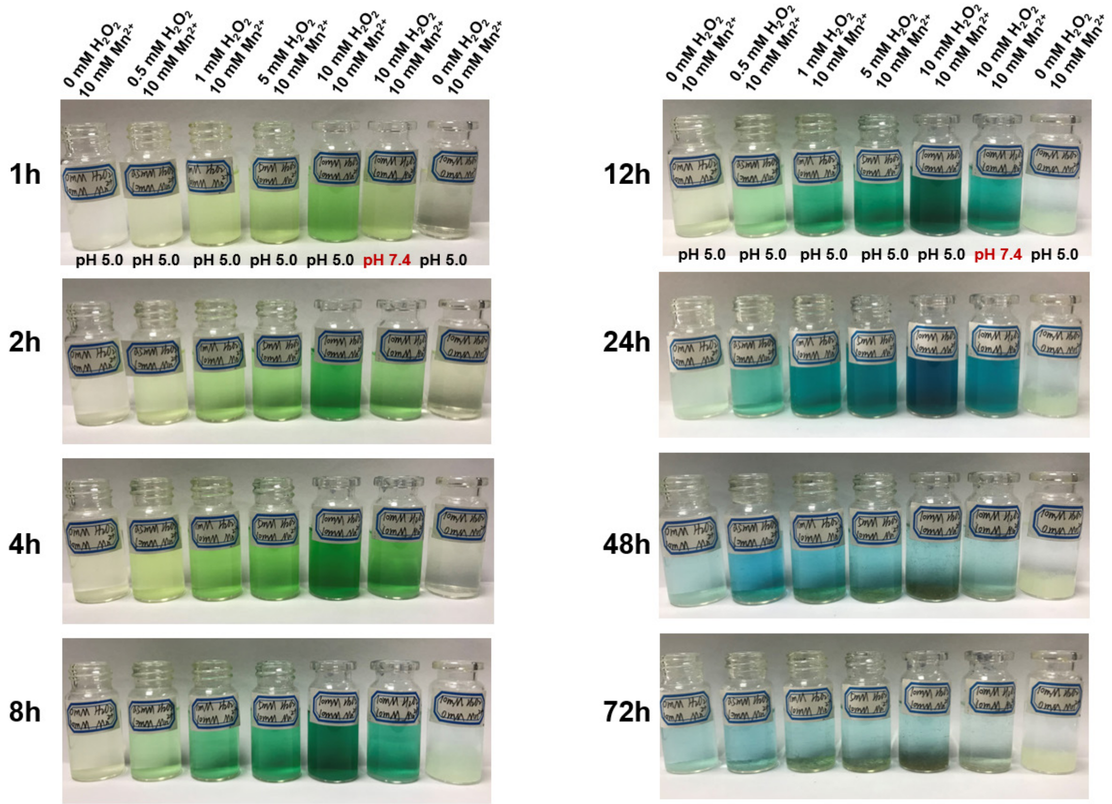

Figure S9. The color changes of Mn^{2+} and H_2O_2 after incubation with TMB for different times.

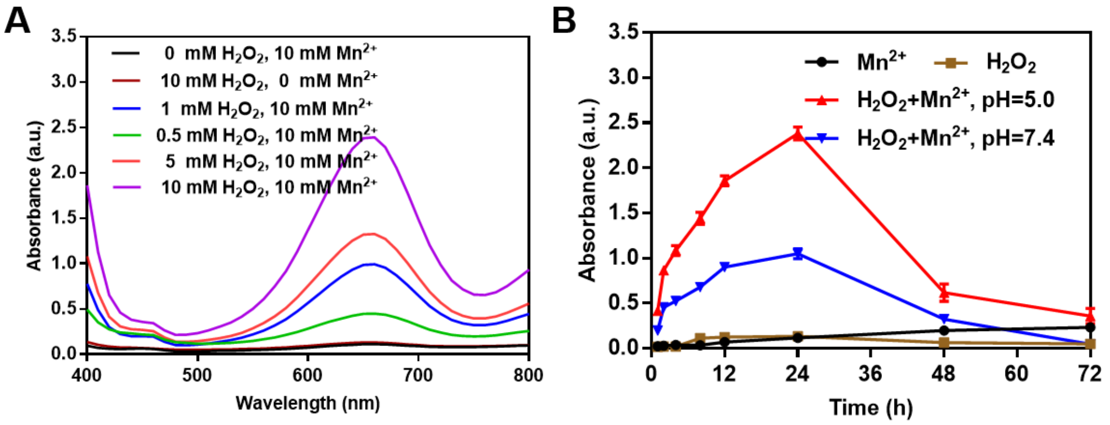

Figure S10. UV-vis spectra of TMB solutions after co-incubation for 24 h with different concentrations of H_2O_2 and Mn^{2+} (A). Absorbance changes of TMB solution incubated with H_2O_2 and Mn^{2+} during 72 h (B).

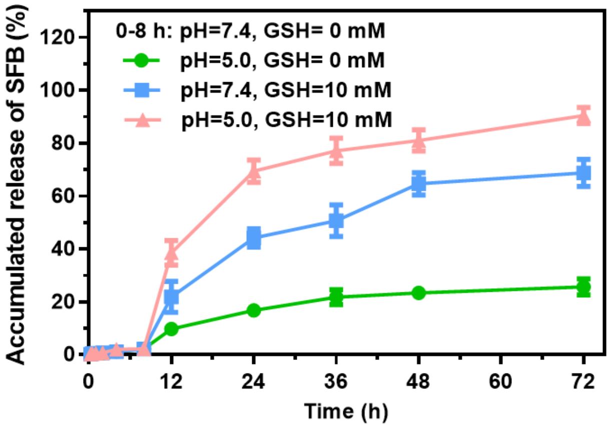

Figure S11. *In vitro* release profile of MnMSN@SFB in PBS at different pH with/without GSH.

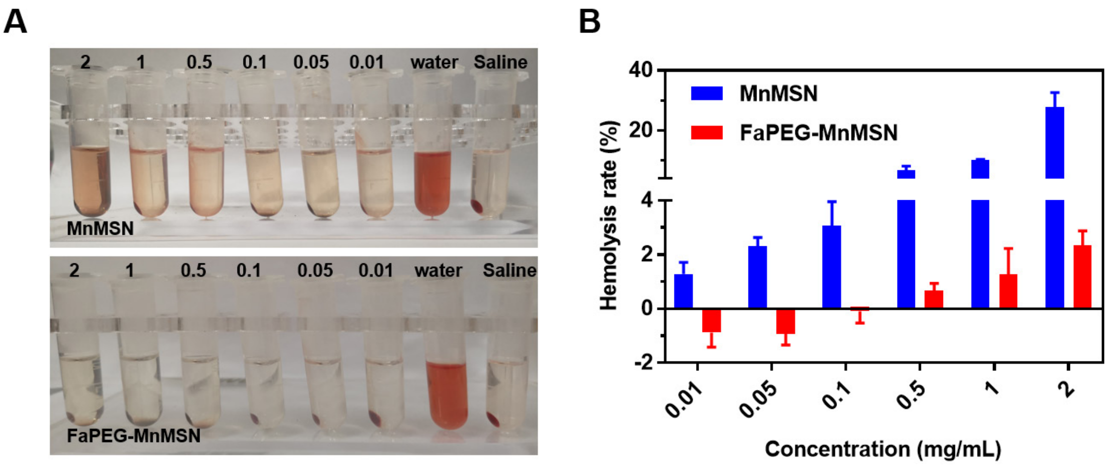

Figure S12. Hemolytic toxicity of MnMSN and FaPEG-MnMSN.
